# Expert consensus on the use of oral antiseptics in oral surgery: Evidence-based clinical practice guidelines

**DOI:** 10.4317/medoral.28160

**Published:** 2026-06-28

**Authors:** Alba Sánchez Torres, María Baus Domínguez, Octavi Camps Font, Celia Carrillo García, Carlos Cobo Vazquez, Jorge Toledano Serrabona, Marta García García

**Affiliations:** 1 Faculty of Medicine and Health Sciences, University of Barcelona, Barcelona, Spain. IDIBELL Institute, Barcelona, Spain; 2 Department of Stomatology, Faculty of Dentistry, University of Sevilla, Sevilla, Spain; 3 Faculty of Health Science, Department of Dentistry, European University of Valencia, Valencia, Spain; 4 Department of Clinical Specialities, Faculty of Dentistry, Complutense University of Madrid, Madrid, Spain

**Keywords:** Chlorhexidine, oral antiseptics, dentoalveolar surgery, implantology, postoperative complications.

## Abstract

**Background:**

Antiseptic management in oral surgery remains heterogeneous, with no standardised, procedure-specific protocols guiding the perioperative use of agents such as chlorhexidine (CHX) or cetylpyridinium chloride (CPC). This consensus document aims to integrate the best available evidence with structured expert judgement to generate evidence-based clinical practice guidelines for the use of oral antiseptics across dentoalveolar and implant surgical scenarios.

**Materials and Methods:**

A structured two-round expert consensus process was employed, combining a systematic literature search of PubMed/MEDLINE with a structured expert panel discussion conducted in December 2025. Seven oral surgeons and implantologists from four leading Spanish universities participated. Consensus was organised around five thematic blocks regarding the use of oral antiseptics: indications and safety, preoperative preparation, postoperative care, management of complications, and future research priorities.

**Results:**

Following independent duplicate screening, 69 studies were included in the qualitative synthesis. CHX 0.12-0.2% (in monotherapy or in combination with CPC) was established as the reference perioperative antiseptic, with evidence supporting its efficacy in reducing postoperative complications (RR=0.66; 95% CI 0.55 to 0.80). A risk-stratified postoperative protocol was formulated, incorporating gel, rinse, and spray formulations adapted to individual patient profiles. Povidone-iodine and CPC were identified as evidence-supported alternatives. Complication-specific recommendations were generated for wound dehiscence, alveolar osteitis, and localised postoperative infection, ranging from conservative antiseptic irrigation and intra-alveolar gel application to escalating surgical and systemic antibiotic management based on severity. All recommendations were explicitly classified as evidence-based, expert consensus-derived, or a combination, reflecting the variable quality of supporting data across clinical scenarios.

**Conclusions:**

CHX constitutes the gold standard oral antiseptic for perioperative use in oral surgery. The expert panel recommends CHX+CPC combination formulations (e.g., 0.12% CHX+0.05% CPC) as an alternative perioperative antiseptic option, offering comparable antimicrobial efficacy with

## Introduction

Bacterial contamination in the oral surgical field remains a persistent challenge despite advances in aseptic protocols and antibiotic prophylaxis. During tooth extraction or implant placement, the oral microbiota becomes dispersed as aerosols and can be deposited into fresh surgical wounds. This disrupts the ecological balance and exposes tissues that cannot yet mount effective defences against biofilm formation [1]. Postoperative infections occur in approximately 2% of oral surgical procedures, although this rate varies according to the type of intervention and patient-related factors [2]; moreover, infection represents only one manifestation of bacterial overgrowth, which also includes delayed wound healing, increased postoperative pain, and alveolar osteitis following tooth extraction [3,4].

Chlorhexidine (CHX) has been established as the gold standard oral antiseptic for more than five decades, owing to its broad-spectrum antimicrobial activity, substantivity (prolonged retention on oral surfaces), and proven clinical efficacy in reducing plaque accumulation and postoperative complications [5]. Cetylpyridinium chloride (CPC) has emerged as an alternative or complementary agent with a potentially lower incidence of adverse effects, particularly tooth staining [6]. Despite extensive investigation, clinical protocols remain highly heterogeneous in concentration, frequency, duration of use, and formulation.

There is a need for evidence-based clinical practice guidelines to standardise antiseptic use in oral surgery, with particular emphasis on dentoalveolar surgery and implantology. Current prescribing patterns vary notably among clinicians, and despite widespread antiseptic use, clearly defined protocols tailored to specific procedures, individual patient risk profiles, and the management of complications are lacking. The present consensus document aims to integrate the best available scientific evidence with expert clinical judgement to generate practical, scenario-specific recommendations that support the safe and effective use of oral antiseptics in routine oral surgical practice.

## Materials and Methods

This consensus statement was developed through a modified Delphi methodology conducted in Barcelona on December 12, 2025, involving a panel of seven oral surgeons and implantologists affiliated with four leading Spanish universities. The process was grounded in a systematic literature review performed in PubMed/MEDLINE using a structured search strategy combining MeSH terms and free-text keywords encompassing antiseptic agents and a broad range of oral surgical procedures. Eligible studies included randomised controlled trials, controlled clinical trials, cohort studies, and clinically relevant systematic reviews or meta-analyses, restricted to populations undergoing oral surgical procedures with perioperative or postoperative antiseptic interventions. Studies focused on periodontal maintenance, laboratory experiments, or isolated case reports were excluded.

Two independent reviewers screened all records and extracted data using a standardised template, resolving discrepancies by consensus, with additional literature identified through manual searching. Each included study was classified according to its design using the SIGN study design algorithm, and the corresponding SIGN methodology checklist was applied: Checklist 1 for systematic reviews and meta-analyses, Checklist 2 for randomised controlled trials, Checklist 3 for cohort studies, and Checklist 5 for non-randomised controlled studies, the latter following the modified RCT checklist protocol with items 1.2 through 1.4 omitted and a maximum assignable evidence level of 2+. Narrative reviews, expert communications, and non-comparative case series, which fall outside the scope of the SIGN checklist framework, were assigned SIGN evidence levels 4 and 3, respectively, without formal checklist appraisal. The results of these assessments are presented in Supplementary Table 1 (https://www.medicinaoral.com/medoralfree01/28160/Suppl1.pdf) and were explicitly considered when formulating the strength of each consensus recommendation. Studies with low SIGN evidence level were assigned lower evidential weight in the deliberation process. Based on this, each recommendation was assigned a GRADE-inspired classification reflecting both the strength of the recommendation and the quality of the supporting evidence: Grade A (Strong, supported by consistent moderate-to-high quality evidence); Grade B (Moderate, supported by moderate quality evidence or strong indirect evidence); and Grade C (Weak/Conditional, based on low or very low quality evidence or predominantly expert consensus). The expert panel discussion was organised into five predefined thematic blocks covering indications, preoperative and postoperative protocols, complication management, and future research priorities, with deliberations explicitly distinguishing evidence-based recommendations from expert opinion where data were limited or conflicting. A detailed description of the methodology is provided in the Supplementary Methods (https://www.medicinaoral.com/medoralfree01/28160/Suppl1.pdf).

## Results

The initial search identified 582 articles. After title and abstract screening, the full text of 111 articles was read by two researchers, resulting in the inclusion of 52 original studies (Cohen's kappa=0.72). An additional 17 publications were added through manual searching, yielding a total of 74 studies for qualitative synthesis: Randomised or controlled clinical trials (n=37); case series (n=2); *in vitro* studies (n=1); narrative reviews/expert communications (n=10); cohort/case control studies (n=10); and systematic/umbrella reviews or meta-analyses (n=15) (full list of articles included can be found in Supplementary Table 1 (https://www.medicinaoral.com/medoralfree01/28160/Suppl1.pdf)

*Indications, contraindications, and safety of antiseptics*

*Indications*

CHX is useful in both the preoperative and early postoperative phases to maintain a low bacterial load, reduce the likelihood of infectious complications, and facilitate the integration of grafts and biomaterials in guided bone regeneration procedures [7]. Its principal indications include reduction of intraoral bacterial load, prevention of postoperative infection, control of biofilm accumulation when mechanical plaque control is compromised, support of optimal wound healing conditions, and adjunctive use alongside routine hygiene measures [6,8]. Systematic reviews and meta-analyses indicate that perioperative CHX use significantly improves wound healing after oral surgery when compared to controls where CHX was not employed (RR=0.66, 95% CI=0.55-0.80, *p<*0.001, I2=85.9%) [9]. Both gel and rinse formulations are effective, although the enhanced bioadhesive properties of gels may provide a modest advantage by prolonging contact time at the surgical site [9] (Grade A). The combination of 0.12% CHX and 0.05% CPC produces a synergistic effect that achieves complete inhibition of bacterial growth in saliva, demonstrating superior biofilm control compared with CHX monotherapy [10-12].Although the use of oral CHX antiseptics may benefit all patients by reducing bacterial load [13-15], the consensus panel emphasised that antiseptic use is particularly recommended in patients at increased risk of postoperative complications [16-18], even though high-quality data in these populations are limited. These groups include individuals with diabetes mellitus, immunocompromised patients, smokers, patients with poor baseline oral hygiene, and those undergoing complex procedures characterised by prolonged surgical time or extensive bone manipulation [19]. Use of CHX in pregnant women and children should be restricted to situations with clear clinical justification [8], given that these populations are underrepresented in clinical trials; most studies have included patients aged 15 years or older, and although no specific teratogenic effects have been documented, the limited data in pregnancy mandate cautious use and informed patient consent.

*Contraindications*

The main contraindication to CHX use is documented hypersensitivity to it or to any excipient of the formulation, with clinical manifestations ranging from mild mucosal lesions to severe anaphylaxis, although true CHX allergy is rare in dental practice [7,20]. When CHX is contraindicated, povidone-iodine (PVP-I) at 0.5-1% represents the best-supported alternative, offering rapid bactericidal activity albeit with a residual effect inferior to that of CHX [21]. CPC, essential oil-based mouthrinses, super-oxidised solutions, and cetrimide are additional options, though with more limited supporting evidence in oral surgery [22] (Grade B). 

*Safety*

Tooth staining is the most frequent adverse effect associated with CHX, arising from the interaction between CHX and dietary chromogens, particularly those present in coffee, tea, red wine, and tobacco; its frequency and intensity correlate directly with treatment duration and CHX concentration [5,8,23]. Discolouration can be mitigated by using formulations incorporating anti-discolouration systems (ADS), anti-staining [24] or whitening toothpastes, and professional prophylaxis. There is moderate quality evidence that the addition of ADS to CHX mouthrinse reduces tooth surface discolouration without compromising the anti-plaque and anti-gingivitis efficacy of CHX when the mouthrinse is used as the sole oral hygiene measure, although ADS does not have a significant effect on tooth staining when CHX is used as an adjunct to mechanical toothbrushing [25]. Clinicians should therefore be aware that the anti-discolouration benefit of ADS-containing CHX formulations applies specifically to regimens in which the mouthrinse is the primary or sole oral hygiene measure. When patients are able to brush normally alongside CHX rinsing during the postoperative period, ADS does not provide a statistically significant reduction in staining, and recommending these formulations primarily on the basis of cosmetic tolerability in brushing patients is not supported by current evidence.

Dysgeusia, reported more frequently with CHX than with CPC, manifests as metallic or bitter taste persisting for several hours after rinsing. Mucosal irritation, burning sensation, and xerostomia occur less frequently but may adversely affect adherence [7]. Parotiditis, although rare, has also been described [26]. The panel recommended distinguishing mild irritative phenomena from true allergic reactions: The former may respond to dose reduction, formulation change (e.g., rinse to gel), concentration adjustment (0.2% to 0.12%), or substitution with CPC, whereas the latter require complete discontinuation (Grade C).

Daily CHX rinsing reduces microbial diversity, with more pronounced changes observed as treatment duration increases, modulating oral microbiome structure and contributing to resolution of dysbiosis. The medium- and long-term ecological consequences of repeated antiseptic courses in patients undergoing frequent surgical interventions or implant maintenance remain insufficiently characterised, representing a priority for future microbiome-focused research [6]. *In vitro* studies have demonstrated cytotoxic effects of CHX on several oral cell types [27], including gingival fibroblasts, at clinically relevant concentrations [28], raising theoretical concerns about interference with wound healing. However, three dimensional culture models and clinical data indicate that CHX at 0.12-0.2%, used for short courses of 7-14 days, does not impair healing. [29,30]. The panel concluded that *in vitro* cytotoxicity findings, largely derived from two-dimensional monolayer systems that lack the structural and biological complexity of intact oral tissues, cannot be directly extrapolated to the clinical setting. Of note, the cytotoxic concentration thresholds reported for CHX in monolayer cell cultures are among the lowest recorded for any clinically used antiseptic, which warrants continued pharmacovigilance particularly in the context of high-frequency or long-duration regimens, even though these perioperative courses (7-14 days) have not been associated with clinically significant impairment of healing *in vivo* [31] (Grade C). Prolonged continuous CHX exposure exceeding six months has been associated with accumulation of p-chloroaniline, a metabolite with mutagenic potential in preclinical models; accordingly, therapy should be restricted to defined short courses, which show an excellent safety profile with no evidence of significant metabolite accumulation [7].

*Preoperative preparation protocols for dentoalveolar surgery and implantology*

*Standard preprocedural rinsing protocol*

Antiseptic rinses significantly reduce intraoral bacterial counts, with a more pronounced effect on anaerobes. Cetrimide appears to be the most effective and persistent agent in this context, followed by CHX, whose substantivity provides superior residual activity, sustained for up to 7 hours on salivary flora, making it particularly advantageous in procedures lasting more than one hour [32,33]. The recommended preprocedural protocol consists of 10-20 mL of CHX 0.12-0.2% for 30-60 seconds immediately before surgery [34]. Comparative studies suggest that both concentrations provide similar clinical efficacy in reducing bacterial load (0.12% delivering approximately 18mg per rinse at 15mL, 0.2% delivering around 20mg at 10mL), while 0.12% tends to be better tolerated owing to fewer or milder adverse effects [35] (Grade B). The expert panel further supports that CHX+CPC combination formulations (e.g., 0.12% CHX+0.05% CPC, 10-20mL for 30-60 seconds) constitute an alternative preprocedural option applicable to all patients.

Some protocols recommend initiating CHX rinses several days before surgery in very high-risk patients (e.g., poorly controlled diabetes or severe immunosuppression); however, this approach lacks support from clinical trials and is based largely on empirical practice. The panel concluded that a single supervised chairside rinse immediately prior to surgery provides the principal clinical benefit by maximising bacterial load reduction at the time of the procedure (Grade C).

*Alternative preprocedural antiseptics*

PVP-I is a well-established antiseptic widely used as a preprocedural rinse due to its broad-spectrum activity against bacteria, viruses, and fungi. It has a rapid onset of action and an antimicrobial effect lasting 2-4 hours [32]. Effective concentrations range from 0.5 to 1% with recommended rinse times of 30-60 seconds [21], and studies have demonstrated marked reduction of microbial and viral loads, including SARS-CoV-2, after contact times as brief as 15 seconds [36]. Its pharmacokinetic profile makes it particularly suitable for procedures lasting up to one hour, where rapid onset is advantageous and the relatively brief duration of effect is less clinically relevant [32]. PVP-I is contraindicated in individuals with iodine allergy, thyroid disease, or during pregnancy and lactation, and may induce a reversible brownish discolouration of mucosa or teeth. CPC (0.05-0.075%, 30-60 seconds) represents an effective alternative for patients unable to tolerate CHX [34], although its lower substantivity makes CHX preferable for procedures exceeding 1-2 hours. Beyond its antibacterial properties, CPC has demonstrated virucidal activity against enveloped respiratory viruses, including SARS-CoV-2, influenza A, and respiratory syncytial virus, through disruption of the viral lipid envelope [37]. Of note, formulations combining CPC+CHX at reduced concentrations (e.g., 0.12% CHX + 0.05% CPC or 0.05% CHX + 0.05% CPC) have shown equivalent plaque-inhibitory efficacy to standard 0.2% CHX mouthrinses while improving taste tolerance and reducing adverse effects [38]. Essential oil-based mouthrinses [39] and super-oxidised solutions are supported by less robust evidence in oral surgery, and are generally reserved for situations in which CHX or PVP-I are contraindicated or poorly tolerated [22] (Grade B).

*Types of procedures*

Although no differentiated, standardised antiseptic protocols have been defined for individual procedures [34], the panel considered preprocedural antiseptic rinsing a universal infection control measure applicable to all oral surgical interventions, consistent with guidance from professional organisations [34]. The supervised preprocedural rinse is a low-cost, low-risk intervention with documented efficacy in reducing intraoral bacterial load and aerosol contamination, and its minimal time requirement and negligible cost relative to the potential reduction in postoperative complications support its routine implementation [40].

Although the evidence base for procedure-specific differentiation remains limited, the expert panel considered it clinically meaningful to stratify antiseptic protocols according to procedural complexity and patient systemic risk. For simple dentoalveolar surgery in systemically healthy patients, a single chairside preprocedural rinse is considered sufficient. For moderate-complexity procedures (impacted third molar surgery, routine implant placement, minor pre-prosthetic surgery), the same preprocedural protocol is recommended, with postoperative antiseptic therapy initiated within 24 hours. For complex regenerative or implant procedures (bone augmentation, guided bone regeneration, sinus floor elevation, soft tissue grafts) and for all procedures in high-risk patients (poorly controlled diabetes, immunosuppression, heavy smoking, prior jaw radiotherapy), a more intensive perioperative protocol including preoperative rinsing, structured postoperative antiseptic therapy for a minimum of 7 days, and clinical follow-up at 7 days is recommended (Grade B).

*Postoperative care protocols*

*Standard postoperative antiseptic regimen and risk-stratified protocol adaptation*

Based on the available evidence, the expert panel agreed on a standard postoperative antiseptic regimen for uncomplicated oral surgical procedures according to a preliminary risk stratification (Figure 1). CHX, alone or in combination with CPC, must not be considered a substitute for antibiotic prophylaxis when this is otherwise indicated (Grade C). Protocol adaptation should incorporate surgical risk determinants, including duration and complexity of the procedure, extent of bone manipulation, degree of soft tissue trauma, and quality of primary closure. In healthy individuals with excellent oral hygiene undergoing minor, uncomplicated procedures (e.g., single tooth extraction with intact socket walls and tension-free primary closure), some studies suggest that mechanical irrigation with saline may be sufficient, and routine antiseptic use may be unnecessary [41] (Grade C).

*Alternatives in patients with low tolerance*

Several antiseptic agents and formulations can be considered when standard CHX is contraindicated or poorly tolerated. These include CPC, warm saline irrigation, PVP-I, and super-oxidised solutions. Additionally, formulations combining CHX with other agents such as αbisabolol, hypericum oil, olive oil, or hydrogen peroxide plus sodium hyaluronate are available. However, the evidence supporting their efficacy in oral surgery is relatively limited and, in general, their antimicrobial and biofilm-controlling properties do not reach the level demonstrated for CHX [42,43], reserving antimicrobial rinses for situations where systemic or local risk factors substantially increase the probability of complications [44]. For patients who present tolerance issues with conventional CHX, bioadhesive or combination formulations such as CHX with CPC, chitosan, dexpanthenol, allantoin, eugenol, or hydrogen peroxide plus sodium hyaluronate can offer enhanced support for tissue repair while potentially extending antimicrobial action in compromised patients. When genuine adverse reactions to CHX occur, CPC, hyaluronic acid-based products, or essential oil-based mouthrinses [45] provide reasonable alternatives, although with the understanding that antimicrobial potency may be somewhat lower than with CHX.

*Precautions during postoperative care*

Liquid rinses are particularly indicated when the surgical site should not be directly manipulated, as gentle rinsing enables antiseptic delivery without mechanical trauma to healing tissues. Patients must be instructed to rinse gently rather than vigorously, because intense swishing can disrupt the blood clot or dislodge sutures. CHX gels (usually 0.2%) afford advantages for localised application and prolonged tissue contact [43,46,47]. They are especially useful for intra-alveolar application after dental extraction, where a single application can diminish the incidence of alveolar osteitis and reduce pain. For postoperative use, gels may be placed with a clean finger or a cotton swab to accessible surgical sites. CHX sprays are an alternative for patients who cannot rinse effectively or when direct access is limited; they are useful when the surgical site should not be disturbed, yet liquid rinses cause discomfort or are difficult to manage. Although sprays are less commonly prescribed in routine practice, the panel considered them a valuable option in selected patients (Grade C).

When standard CHX regimens result in adverse effects that compromise adherence, practical modifications of formulation and concentration provide viable solutions. For patients concerned about tooth staining during the typical 7-14day postoperative course, CHX formulations containing ADS and anti-staining toothpastes offer an evidence-based strategy that maintains antimicrobial efficacy while reducing cosmetic concerns. The 7 day minimum covers the critical early phase of wound healing when mechanical plaque control is most compromised and bacterial challenge is highest. Patients should be informed that transient discolouration may still occur, although professional prophylaxis usually removes staining effectively, and adjunctive use of stain-removing toothpaste further reduces this problem. When dysgeusia is the limiting factor, particularly in individuals with marked taste sensitivity who might discontinue therapy, switching from a full-mouth liquid rinse to localised gel application allows preservation of antimicrobial coverage while substantially reducing exposure of the entire oral cavity to the bitter taste of CHX.

For patients with more pronounced intolerance, substitution with CPC 0.05-0.075% is a rational alternative that generally results in less dysgeusia and less tooth staining while offering satisfactory antimicrobial efficacy for postoperative care. When used as a twice daily rinse for at least 7 days, CPC can provide adequate antiseptic benefit without the tolerability issues that could lead to premature discontinuation. The selection among these strategies should be individualised according to the patient’s specific tolerance profile and the relative importance assigned to each adverse effect in terms of quality of life (Grade B).

*Management of postoperative complications*

The evidence regarding complication management recommendations is substantially weaker than that available for prophylactic antiseptic use. Direct head-to-head comparisons of antiseptic protocols for wound dehiscence, alveolar osteitis, and postoperative infection are absent from or poorly represented in the indexed literature. The recommendations in this section are therefore based predominantly on structured expert consensus, physiological and pharmacological reasoning, and indirect extrapolation from more general wound care evidence. 

*Wound dehiscence*

Wound dehiscence, defined as partial or complete separation of sutured wound margins with exposure of the underlying tissues, is a frequent complication after bone regeneration procedures, particularly those involving bone augmentation, membrane placement, or soft tissue grafting. The panel distinguished between minor dehiscence, characterised by small exposure areas (typically ≤5 mm) without suppuration or overt signs of infection, and major dehiscence, defined by extensive exposure, clinical evidence of infection, or involvement of grafts and membranes. For minor dehiscence, conservative management represents the treatment of choice [48]. Management includes meticulous oral hygiene, gentle irrigation with CHX solution (monotherapy or in combination with CPC), and application of CHX gel twice daily directly to the exposed area. Patients must be instructed to maintain rigorous plaque control around the dehiscence while avoiding trauma to the affected tissues. Weekly follow-up is recommended to monitor healing and allow early detection of infection (Grade C).

*Alveolar osteitis (dry socket)*

Alveolar osteitis, or dry socket, is characterised by premature disintegration or loss of the blood clot from the extraction socket, resulting in exposed bone and intense pain. Prevention and effective management of alveolar osteitis are essential to reduce postoperative morbidity [49]. The condition typically appears 2-4 days after extraction, presenting as severe, throbbing pain that often responds poorly to standard analgesics and may radiate along the course of the involved nerve. Clinically, the socket appears partially or completely devoid of clot, with exposed alveolar bone and frequently a foul odour and debris in the socket [17]. Management includes gentle irrigation of the socket with CHX to remove debris, CHX gel, analgesics, and regular follow-up until granulation tissue covers the exposed bone [50] (Grade B).

Major complications that require surgical reintervention

The initial approach to suspected postoperative infection should include a thorough clinical assessment to determine its severity and extent. Localised infections without systemic manifestations may respond to an intensified antiseptic regimen, including more frequent CHX (in monotherapy or in combination with CPC) applications and gentle antiseptic irrigation to promote drainage. The surgical area should be evaluated for sutures under excessive tension, which may need to be removed to allow drainage, and for retained foreign bodies that must be eliminated. When signs of infection are more than minimal, or systemic symptoms are present, systemic antibiotic therapy is indicated. Antibiotic choice should consider local resistance patterns and patient characteristics, with amoxicillin, amoxicillin-clavulanate, and clindamycin (in penicillin-allergic patients) being first-line options for odontogenic infections [51].

*Future perspectives and research priorities*

*Novel antiseptic formulations and combinations*

The panel reviewed data on alternative antiseptic agents that may offer efficacy comparable to CHX with fewer adverse effects. Octenidine, olanexidine, and lauroyl arginine ethyl ester (LAE) have shown antimicrobial activity similar to CHX in early studies and are associated with less tooth staining and mucosal irritation in some reports. Nevertheless, clinical evidence specific to oral surgery is still limited, and none of these agents currently has a sufficient evidence base to warrant recommendation as a preferred routine alternative to CHX.

*Controlled-release and localised delivery systems*

Bioadhesive drug-delivery platforms, including hydrogels, mucoadhesive films, and chlorhexidine-impregnated chips, represent a fundamentally different strategy for antiseptic administration that seeks to achieve sustained antimicrobial action with fewer tolerability limitations than conventional rinses [52]. These systems can maintain effective antimicrobial concentrations over hours to days through gradual release of chlorhexidine, avoiding the sharp concentration peaks associated with mouthrinses and reducing related adverse effects such as taste disturbance and staining. By delivering the agent directly to the target site, whether a periodontal pocket, surgical defect, or peri-implant region, these devices focus antimicrobial action where it is most needed while limiting unnecessary exposure of the rest of the oral cavity, thereby reducing systemic adverse effects and improving tolerability. Furthermore, bioadhesive systems can interact with tissues and biofilms, modulating the local biological response and supporting resolution of inflammation. Clinical studies in periodontal and peri-implant indications have shown sustained improvements for 6-12 months, including reduced probing depth, gains in clinical attachment level, decreased pathogenic bacterial counts, and improved soft tissue health. Emerging controlled-release technologies such as nanoparticle formulations, advanced hydrogels, and “intelligent” localised delivery devices remain insufficiently evaluated in medium- and long-term clinical trials. Although preliminary data are promising, broader adoption will depend on robust safety and efficacy evidence in diverse patient populations and surgical contexts. The panel considers controlled-release delivery a promising frontier for optimising antiseptic therapy and recommends prioritising clinical research to clarify its role in oral surgery.

*Clinical research priorities*

The panel identified a clear need for well-designed randomised controlled trials directly comparing CHX with newer or alternative antiseptics (including octenidine, LAE, natural product mouthrinses, and nanoparticle-based formulations) in specific oral surgery settings. Such trials should have adequate statistical power, clinically relevant endpoints (postoperative complications, pain, healing trajectories, and patient-reported quality of life), systematic assessment of adverse effects, and follow-up long enough to capture late complications and long-term safety signals. Special priority should be given to head-to-head comparisons in high-risk procedures, where antiseptic choice may significantly influence outcomes. Study populations should include an adequate representation of high-risk groups (e.g., patients with diabetes, immunosuppression, or heavy smoking) to allow prespecified subgroup analyses examining whether antiseptic performance varies by risk profile. Key questions could include those presented in Table 1.

The *in vitro* cytotoxicity data for CHX and other antiseptics, often generated in two-dimensional monolayer cell cultures, may not adequately reflect clinical reality. The panel therefore called for studies using three-dimensional tissue models and organotypic cultures that more faithfully replicate the architecture and cellular interactions of the oral mucosa. Such models may help reconcile apparent discrepancies between *in vitro* cytotoxicity findings and clinical outcomes.

Microbiome-focused research was also identified as a priority. Studies employing next-generation sequencing are needed to characterise changes in oral microbial communities before and after antiseptic use. Important questions include the duration of CHX-induced microbiome alterations after treatment cessation, the capacity of short courses (7-14 days) to enable rapid re-establishment of commensal flora, and whether targeted strategies (e.g., probiotics or selective antiseptics) might preserve beneficial species while controlling pathogens.

Finally, the panel advocated the creation of registries or prospective cohort studies collecting standardised data on antiseptic regimens, patient risk factors, surgical procedures, and outcomes to link specific practices with clinical results. Such large-scale observational studies of real-world antiseptic use would complement randomised trial data by characterising adherence patterns, adverse event rates in heterogeneous populations, effectiveness in routine practice conditions, and long-term outcomes.

Some limitations of this consensus should be acknowledged. The bibliographic search was restricted to PubMed/MEDLINE. Although this database provides broad coverage of the indexed dental and oral surgery literature and the search was supplemented by an extensive manual search of 17 additional publications, the absence of searches in Embase, Cochrane Library, or CINAHL represents a methodological limitation that may have resulted in the omission of a minority of eligible studies. Future updates of this guideline should incorporate multi-database searches to further strengthen the comprehensiveness of the evidence base. The included evidence predominantly derives from adult populations capable of performing standard rinsing protocols, limiting the generalisability of these recommendations. Populations insufficiently represented include paediatric patients, individuals with physical or intellectual disabilities, patients with severe dysphagia or dementia, and those with profound xerostomia secondary to radiotherapy. In these groups, age-appropriate or localised formulations (gel, spray) and modified delivery strategies should be considered, and specialist guidance sought where appropriate.

*Summary of expert panel recommendations*

To facilitate clinical implementation, the expert panel formulated the following set of consensus recommendations, organised according to the thematic blocks addressed in this document. Each recommendation is classified according to its evidentiary basis: Evidence-based (EB), supported by data from randomised controlled trials, systematic reviews, or meta-analyses; expert consensus (EC), derived primarily from clinical experience and panel deliberation in areas where robust trial data are lacking; or a combination of both (EB/EC), where available evidence was supplemented by expert interpretation (Table 2).

## Conclusions

CHX emerges as the reference oral antiseptic in dentoalveolar surgery and implantology, supported by substantial evidence showing that short, welldefined perioperative regimens effectively reduce biofilm, postoperative pain, and complications, particularly in patients with systemic or local risk factors. Its use should be carefully tailored to individual risk, with attention to contraindications and frequent adverse events such as staining and dysgeusia, which can often be mitigated through dose adjustment, selection of gels or sprays, incorporation of antidiscolouration systems or anti-staining solutions, or rational substitution with agents such as CPC or PVPI when true intolerance or allergy is present. Preoperative antiseptic rinsing with CHX 0.12-0.2% immediately before surgery is reinforced as a simple, lowcost intervention with clear microbiological and clinical benefits and should be considered a universal measure in oral surgery, while CHX+CPC combination formulations (e.g., 0.12% CHX+0.05% CPC) constitute an equivalent perioperative option with CHX monotherapy, offering comparable antimicrobial efficacy. Alternative agents such as PVPI, CPC, essential oils, or super-oxidised solutions find their place in shorter procedures, in situations of intolerance, or when clinically required. Postoperatively, a riskstratified approach is recommended: Routine 7day CHX rinses for most procedures, more conservative salinebased regimens for healthy, lowrisk patients with excellent hygiene, and tailored use of gels, sprays, bioadhesive combinations, CPC, or hyaluronic acid-containing formulations to preserve adherence and comfort without sacrificing antiseptic control.

In the setting of complications, antiseptics are essential adjuncts but cannot replace surgical judgement: Minor wound dehiscence and localised infection frequently resolve with meticulous hygiene, intensified CHX irrigation, and localised gel application, whereas extensive dehiscence, membrane or graft exposure, and spreading infection require timely debridement, reclosure when feasible, or guided healing by secondary intention together with appropriately selected systemic antibiotics. 

## Supplementary Material

Supplementary material 1

## Figures and Tables

**Table 1:** Table 1: Priority clinical research questions for the optimization of antiseptic protocols in oral surgery.

Question	What is the optimal CHX concentration (0.12% vs. 0.2%) for preprocedural rinsing in terms of bacterial reduction balanced against adverse effects?
	What volume (10mL vs. 15mL vs. 20mL) and duration (30 seconds vs. 60 seconds vs. longer) of preprocedural rinsing maximises antimicrobial benefit?
	How does saline irrigation compare to antiseptic rinsing in low-risk patients with excellent oral hygiene?
	Does multi-day preprocedural home rinsing (e.g. 3-7 days before surgery) provide additional benefit over single chairside rinse immediately before the procedure?

**Table 2:** Table 2: Summary of recommendations.

**Indications, contraindications, and safety**	R1. CHX is the reference antiseptic agent in oral surgery. Its principal indications include reduction of the intraoral bacterial load, prevention of postoperative infection, control of biofilm accumulation when mechanical plaque control is compromised, and support of optimal wound healing conditions. [EB | Grade A]
	R2. Antiseptic use is particularly indicated in patients at increased risk of postoperative complications, including those with diabetes mellitus, immunosuppression, active smoking habit, poor baseline oral hygiene, and those undergoing complex or prolonged surgical procedures. [EC | Grade C]
	R3. The use of CHX in pregnant women and children should be restricted to situations with clear clinical justification, given the limited evidence available in these populations. [EC | Grade C]
	R4. Documented hypersensitivity to CHX or any excipient of the formulation constitutes the t contraindication. When CHX is contraindicated, PVP-I at 0.5-1%, CPC, or essential oil-based mouthrinses are acceptable alternatives, although with lower levels of supporting evidence. [EB | Grade B]
	R5. Postoperative CHX therapy (in monotherapy or in combination with CPC) should be restricted to short, well-defined courses of 7-14 days. Chronic continuous use exceeding six months is discouraged owing to the potential accumulation of metabolites with mutagenic potential. [EB | Grade B]
	R6. Clinicians should distinguish mild irritative phenomena (manageable by dose reduction, formulation change, or substitution with CPC) from true allergic reactions, which require complete discontinuation of CHX. [EC | Grade C]
**Preoperative preparation protocols**	R7. Preprocedural rinsing with CHX 0.12-0.2% (10-20 mL for 30-60 seconds; alone or in combination with CPC) immediately before surgery is recommended as a universal infection control measure applicable to all oral surgical interventions. [EB | Grade B]
	R8. CHX + CPC combination formulations (e.g., 0.12% CHX + 0.05% CPC, 10-20 mL for 30-60 seconds) provide antimicrobial efficacy equivalent to standard 0.2% CHX monotherapy and represent an alternative first-line preprocedural option applicable to all patients. [EB/EC | Grade C]
	R9. A single supervised chairside rinse immediately prior to surgery provides the principal clinical benefit. Multi-day preoperative home rinsing is not supported by current trial evidence and should not be adopted as routine practice. [EB/EC | Grade C]
	R10. For procedures lasting less than one hour, PVP-I (0.5-1%) represents a suitable alternative owing to its rapid onset of action. CPC as monotherapy (0.05-0.075%, 30-60 seconds) remains appropriate when CHX-containing formulations are contraindicated or not tolerated, although its lower substantivity makes CHX-based options preferable for procedures exceeding 1-2 hours. [EB | Grade B]
**Postoperative care protocols**	R11. The standard postoperative antiseptic regimen consists of CHX 0.12-0.2% (monotherapy or combination with CPC) mouthrinse twice daily for a minimum of 7 days and up to 14 days. Protocol duration should be adapted according to patient risk stratification, surgical complexity, extent of bone manipulation, and quality of primary wound closure. [EB/EC | Grade B]
	R12. In healthy patients with excellent oral hygiene undergoing minor, uncomplicated procedures with intact socket walls and tension-free primary closure, mechanical irrigation with saline may be sufficient for postoperative care, and routine antiseptic use may be unnecessary. [EC | Grade C]
	R13. Patients must be instructed to rinse gently to avoid clot disruption or suture dislodgement. CHX gel (0.2%) is indicated for localised application and prolonged tissue contact; CHX sprays constitute an alternative for patients who cannot rinse effectively. [EB/EC | Grade C]
	R14. CHX + CPC combination formulations provide antimicrobial efficacy equivalent to standard CHX 0.2% mouthrinses and should be considered an alternative first-line postoperative antiseptic in any patient, with particular advantage in those prone to dysgeusia or tooth staining. [EB/EC | Grade C]
	R15. For patients with staining concerns, CHX formulations containing an ADS together with anti-staining toothpastes are recommended. For dysgeusia, switching from liquid rinse to localised gel application reduces oral cavity exposure. CPC 0.05-0.075% twice daily for at least 7 days remains a rational alternative when CHX containing formulations cannot be used. [EB/EC | Grade B]
	R16. CHX must not be considered a substitute for antibiotic prophylaxis when this is otherwise indicated. [EC | Grade C]
**Management of postoperative complications**	R17. Minor wound dehiscence (≤5 mm, no suppuration): conservative management with meticulous oral hygiene, gentle CHX irrigation, and CHX gel application twice daily to the exposed area, with weekly follow-up. Major dehiscence with infection or graft/membrane exposure requires surgical reintervention. [EC | Grade C]
	R18. Alveolar osteitis: gentle socket irrigation to remove debris, placement of eugenol or CHX gel, analgesic therapy, and regular follow-up until granulation tissue covers the exposed bone. [EB/EC | Grade B]
	R19. Suspected postoperative infection: initial intensification of the antiseptic regimen with more frequent CHX applications and gentle antiseptic irrigation. Systemic antibiotics (amoxicillin, amoxicillin-clavulanate, or clindamycin in penicillin-allergic patients) are indicated when signs exceed minimal local involvement or systemic symptoms are present. [EB/EC | Grade C]

ADS: Anti-discoloration system; CPC: Cetylpyridinium chloride; CHX: Chlorhexidine; EB: Evidence-based; EC: Expert consensus; EB/EC: Evidence supplemented by expert consensus; PVP-I: Povidone-iodine. Recommendations are classified according to a simplified GRADE-inspired framework: Grade A (Strong) - strong recommendation supported by consistent, moderate-to-high quality evidence; Grade B (Moderate) - moderate recommendation based on moderate quality evidence or strong indirect evidence; Grade C (Weak/Conditional) - conditional recommendation based on low or very low quality evidence, predominantly expert consensus, or significant heterogeneity in the available data. Evidence quality levels reflect the aggregate risk of bias across the supporting primary studies and systematic reviews as assessed by RoB 2, ROBINS-I, and modified AMSTAR-2.

**Figure 1:**
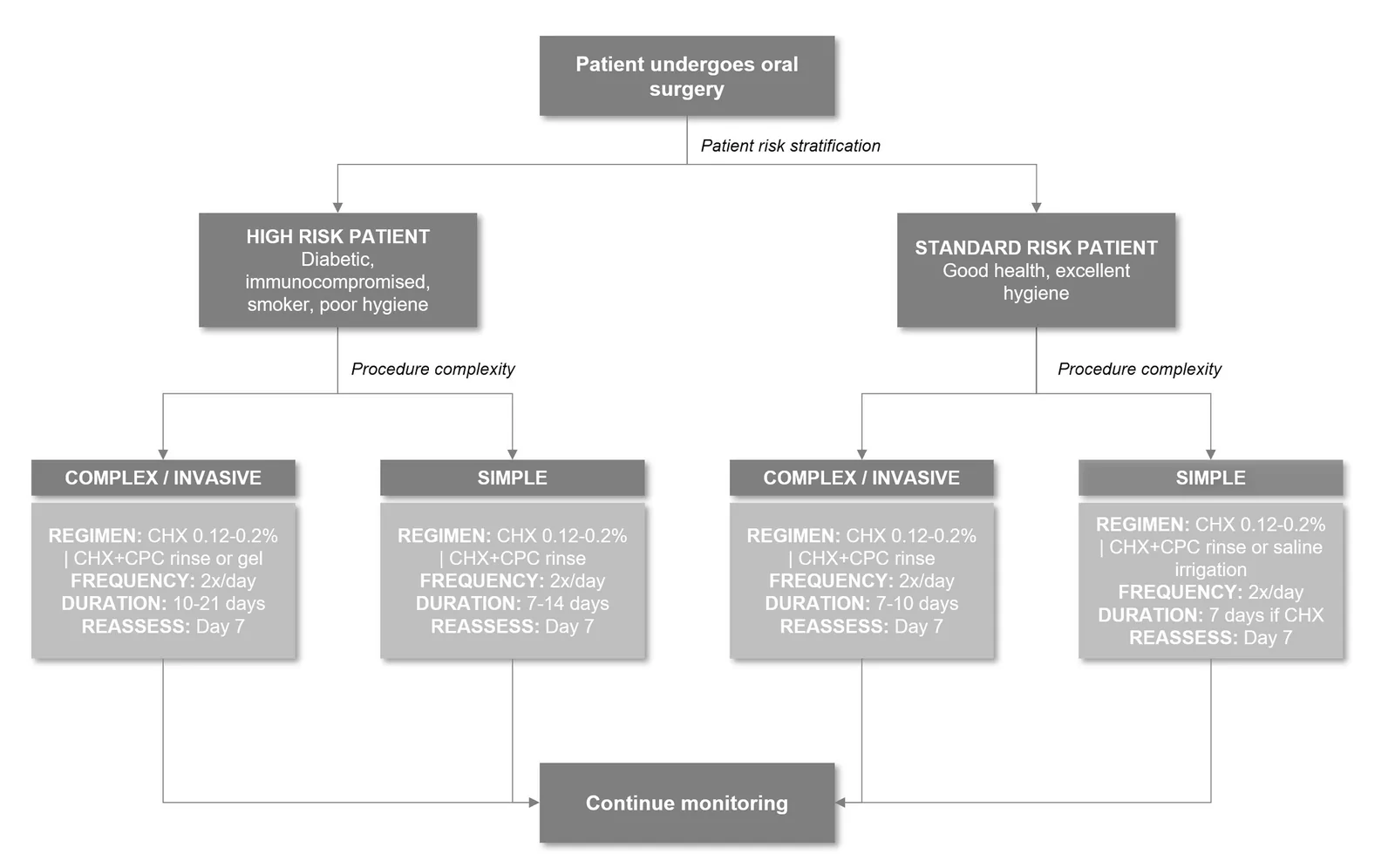
Fig. 1: Postoperative antiseptic risk protocol. CHX: Chlorhexidine; CPC: Cetylpyridinium chloride.

## Data Availability

None.
