# Supplementary material for: Expert consensus on the use of oral antiseptics in oral surgery: Evidence-based clinical practice guidelines
Source: Med Oral Patol Oral Cir Bucal. 2026 Jun 28;31(5):e716–27. doi: 10.4317/medoral.28160 (PMC13636452; doi:10.4317/medoral.28160)
Supplement: Supplementary file 1 [file medoral-31-05-e716-s001.pdf]

SUPPLEMENTARY METHODS

Consensus development process

The consensus meeting took place in Barcelona (Spain) on December 12, 2025 and the process followed a structured two-round expert consensus process that combined a systematic literature review with structured expert panel discussion. The panel consisted of seven oral surgeons and implantologists from leading Spanish universities (Universitat de Barcelona, Universidad de Sevilla, Universidad Complutense de Madrid, and Universidad Europea de Valencia), all with extensive clinical and research experience.

Systematic literature review

Search strategy

A structured literature search was performed in PubMed/MEDLINE using a predefined strategy combining MeSH terms and free-text keywords related to antiseptic agents and oral surgical procedures, without restrictions on date or language. The search string used was: (chlorhexidine OR CPC OR gel OR oral antiseptic) AND (oral surgery OR tooth extraction OR cyst enucleation OR impacted canine OR micro-screw OR third molar OR apical surgery OR dental implants OR soft tissue graft OR connective tissue graft OR free gingival graft OR bone regeneration) AND (postoperative complications OR surgical site infection OR dehiscence).

Eligibility criteria

Studies were included if they met the following criteria: Population: Patients undergoing any oral surgical procedure or receiving treatment for postoperative complications. Interventions and Comparators: Perioperative or postoperative use of topical, intra-alveolar, or preprocedural oral antiseptics. Outcomes: Postoperative complications, healing parameters, adverse events, or patient-reported outcomes. Study designs: Randomised controlled trials, controlled clinical trials, prospective or retrospective cohort studies, and clinically relevant systematic reviews or meta-analyses. Studies exclusively focused on periodontal or peri-implant maintenance therapy, mucogingival surgery, laboratory or animal experiments, cross-sectional studies, isolated case reports, and small case series lacking robust outcome data were excluded.

Study selection process, data extraction and study classification

Two reviewers (MGG and AST) independently screened all records by title and abstract. Studies considered potentially relevant underwent full-text evaluation. Discrepancies between reviewers were resolved by consensus. Additional articles, including systematic reviews, meta-analyses, umbrella reviews, and other relevant studies, were identified through manual searching. Both reviewers independently extracted data using a standardised template capturing study design, antiseptic agent, surgical procedure, sample characteristics, outcome measures, follow-up period, and reported complications or adverse effects.

A qualitative synthesis was conducted, structured around antiseptic type, route of application, clinical indication, and postoperative outcomes, in accordance with PRISMA 2020 recommendations [1]. Each included study was classified according to its design using the Scottish Intercollegiate Guidelines Network (SIGN) study design algorithm last update [2,3], and the corresponding SIGN methodology checklist was applied: Checklist 1 for systematic reviews and meta-analyses, Checklist 2 for randomised controlled trials, Checklist 3 for cohort studies, and Checklist 5 for non-randomised controlled studies, the latter following the modified RCT checklist protocol with items 1.2 through 1.4 omitted and a maximum assignable evidence level of 2+. Narrative reviews, expert communications, and non-comparative case series, which fall outside the scope of the SIGN checklist framework, were assigned SIGN evidence levels 4 and

3, respectively, without formal checklist appraisal. The results of these assessments are presented in **Supplementary Table 1** and were explicitly considered when formulating the strength of each consensus recommendation. Studies with high risk of bias or low SIGN evidence level were assigned lower evidential weight in the deliberation process.

The strength of each consensus recommendation was graded using a simplified GRADE-inspired framework: Grade A (Strong recommendation) was assigned when high- or moderate-quality evidence consistently demonstrated benefit and the expert panel achieved unanimous or near-unanimous agreement; Grade B (Moderate recommendation) was assigned when evidence was of moderate-to-low quality or when panel agreement, although clear, was not unanimous; Grade C (Conditional/Weak recommendation) was assigned in scenarios where evidence was of low or very low quality, extrapolated from indirect sources, or where clinical benefit was judged plausible but not firmly established. Recommendation grades are explicitly reported alongside each clinical recommendation.

Expert panel discussion structure

The consensus meeting was organised into five thematic blocks with key clinical questions defined a priori, each led by one of the consensus members: (1) indications, contraindications, and safety of antiseptic use; (2) preoperative preparation protocols for dentoalveolar surgery and implantology; (3) postoperative care protocols; (4) management of postoperative complications; and (5) future perspectives and research priorities. Consensus was reached through iterative deliberation, explicitly distinguishing between evidence-based recommendations and expert opinion in areas where data were limited or conflicting.

References

1. Liberati A, Altman DG, Tetzlaff J, Mulrow C, Gøtzsche PC, Ioannidis JPA, et al. The PRISMA statement for reporting systematic reviews and meta-analyses of studies that evaluate health care interventions: explanation and elaboration. J Clin Epidemiol. 2009 Oct;62(10):e1-34. doi:10.1016/j.jclinepi.2009.06.006 PubMed PMID: 19631507.

2. Harbour R, Miller J. A new system for grading recommendations in evidence based guidelines. BMJ. 2001 Aug 11;323(7308):334–6. doi:10.1136/bmj.323.7308.334 PubMed PMID: 11498496; PubMed Central PMCID: PMC1120936.

3. SIGN [Internet]. [Accessed 2026 Apr 6]. Checklists. Available from: https://www.sign.ac.uk/using-our-guidelines/methodology/checklists/

SUPPLEMENTARY TABLES

Supplementary Table 1. Included studies from the systematic literature review.

Full Citation	Study Design	SIGN Tool	Item and response	Overall Risk of Bias / Confidence Rating
**Block 1 · Indications, contraindications, and safety of antiseptics**				
1. Balbuena L, Stambaugh KI, Ramirez SG, Yeager C. Effects of topical oral antiseptic rinses on bacterial counts of saliva in healthy human subjects. Otolaryngol Head Neck Surg. 1998;118(5):625–9.	Non-randomised controlled study	Checklist 5: Controlled Trials (Non-Randomised; items 1.2-1.4 omitted)	1.1 The study addresses an appropriate and clearly focused question Yes<br>1.5 The treatment and control groups are similar at the start of the trial No<br>1.6 The only difference between groups is the treatment under investigation Yes<br>1.7 All relevant outcomes are measured in a standard, valid and reliable way Yes<br>1.8 Percentage of individuals in each arm who dropped out before completion Not reported<br>1.9 All subjects analysed in the groups to which they were randomly allocated (ITT) Can't say<br>1.10 Where the study is carried out at more than one site, results are comparable for all sites Can't say	Low quality (−), SIGN 2−: non-randomised controlled study with high risk of bias
2. Duvall NB, Fisher TD, Hensley D, Hancock RH, Vandewalle KS. The comparative efficacy of 0.12% chlorhexidine and amoxicillin to reduce the incidence and magnitude of bacteremia during third molar extractions: a prospective, blind, randomized clinical trial. Oral Surg Oral Med Oral Pathol Oral Radiol. 2013;115(6):752–63.	Randomised controlled trial	Checklist 2: Controlled Trials	1.1 The study addresses an appropriate and clearly focused question Yes<br>1.2 The assignment of subjects to treatment groups is randomized Yes<br>1.3 An adequate concealment method is used Can't say<br>1.4 The design keeps subjects and investigators 'blind' about treatment allocation Yes<br>1.5 The treatment and control groups are similar at the start of the trial Yes<br>1.6 The only difference between groups is the treatment under investigation Yes<br>1.7 All relevant outcomes are measured in a standard, valid and reliable way Yes<br>1.8 Percentage of individuals in each arm who dropped out before completion Not reported<br>1.9 All subjects analyzed in the groups to which they were randomly allocated (ITT) Can't say<br>1.10 Where the study is carried out at more than one site, results are comparable for all sites Can't say	Acceptable (+), SIGN 1+: randomized controlled trial with low risk of bias.

Full Citation	Study Design	SIGN Tool	Item and response	Overall Risk of Bias / Confidence Rating
3. Plantinga NL, Wittekamp BHJ, Leleu K, Depuydt P, Van den Abeele AM, Brun-Buisson C, et al. Oral mucosal adverse events with chlorhexidine 2% mouthwash in ICU. Intensive Care Med. 2016;42(4):620–1.	Multicenter cluster-randomised study	Checklist 2: Controlled Trials	1.1 The study addresses an appropriate and clearly focused question Yes<br>1.2 The assignment of subjects to treatment groups is randomised Yes<br>1.3 An adequate concealment method is used Yes<br>1.4 The design keeps subjects and investigators 'blind' about treatment allocation Yes<br>1.5 The treatment and control groups are similar at the start of the trial No<br>1.6 The only difference between groups is the treatment under investigation Yes<br>1.7 All relevant outcomes are measured in a standard, valid and reliable way No<br>1.8 Percentage of individuals in each arm who dropped out before completion Not reported<br>1.9 All subjects analysed in the groups to which they were randomly allocated (ITT) Can't say<br>1.10 Where the study is carried out at more than one site, results are comparable for all sites Yes	Low quality (−), SIGN 1−: cluster-randomised controlled trial with high risk of bias.
4. Below H, Assadian O, Baguhl R, Hildebrandt U, Jäger B, Meissner K, et al. Measurements of chlorhexidine, p-chloroaniline, and p-chloronitrobenzene in saliva after mouth wash before and after operation with 0.2% chlorhexidine digluconate in maxillofacial surgery: a randomised controlled trial. Br J Oral Maxillofac Surg. 2017;55(2):150–5.	Randomised controlled trial	Checklist 2: Controlled Trials	1.1 The study addresses an appropriate and clearly focused question Yes<br>1.2 The assignment of subjects to treatment groups is randomised Yes<br>1.3 An adequate concealment method is used Yes<br>1.4 The design keeps subjects and investigators 'blind' about treatment allocation Yes<br>1.5 The treatment and control groups are similar at the start of the trial Yes<br>1.6 The only difference between groups is the treatment under investigation Yes<br>1.7 All relevant outcomes are measured in a standard, valid and reliable way Yes<br>1.8 Percentage of individuals in each arm who dropped out before completion Not reported<br>1.9 All subjects analysed in the groups to which they were randomly allocated (ITT) Can't say<br>1.10 Where the study is carried out at more than one site, results are comparable for all sites Can't say	Acceptable (+), SIGN 1+: randomized controlled trial with low risk of bias
5. da Silveira Teixeira D, de Figueiredo MAZ, Cherubini K, de Oliveira SD, Salum FG. The topical effect of chlorhexidine and povidone-iodine in the repair of oral	Narrative review	No SIGN methodology checklist applicable		

Full Citation	Study Design	SIGN Tool	Item and response	Overall Risk of Bias / Confidence Rating
wounds. A review. Stomatologija. 2019;21(2):35–41.				
6. Palaia G, Tenore G, Tribolati L, Russo C, Gaimari G, Del Vecchio A, et al. Evaluation of wound healing and postoperative pain after oral mucosa laser biopsy with the aid of compound with chlorhexidine and sodium hyaluronate: a randomized double blind clinical trial. Clin Oral Investig. 2019;23(8):3141–51.	Randomised double-blind clinical trial	Checklist 2: Controlled Trials	1.1 The study addresses an appropriate and clearly focused question Yes<br>1.2 The assignment of subjects to treatment groups is randomised Yes<br>1.3 An adequate concealment method is used Can't say<br>1.4 The design keeps subjects and investigators 'blind' about treatment allocation Yes<br>1.5 The treatment and control groups are similar at the start of the trial Yes<br>1.6 The only difference between groups is the treatment under investigation Yes<br>1.7 All relevant outcomes are measured in a standard, valid and reliable way Can't say<br>1.8 Percentage of individuals in each arm who dropped out before completion Not reported<br>1.9 All subjects analysed in the groups to which they were randomly allocated (ITT) No<br>1.10 Where the study is carried out at more than one site, results are comparable for all sites Can't say	Acceptable (+) - SIGN 1+ Well-conducted meta-analyses, systematic reviews of RCTs, or RCTs with a low risk of bias
7. Opstrup MS, Jemec GBE, Garvey LH. Chlorhexidine Allergy: On the Rise and Often Overlooked. Curr Allergy Asthma Rep. 2019;19(5):23.	Narrative review	No SIGN methodology checklist applicable		
8. Tartaglia GM, Tadakamadla SK, Connelly ST, Sforza C, Martín C. Adverse events associated with home use of mouthrinses: a systematic review. Ther Adv Drug Saf. 2019;10:2042098619854881.	Systematic review	Checklist 1: Systematic Reviews and Meta-Analyses	1.1 The research question is clearly defined and inclusion/exclusion criteria are listed Yes<br>1.2 A comprehensive literature search is carried out Yes<br>1.3 At least two people should have selected studies Yes<br>1.4 At least two people should have extracted data Yes<br>1.5 Grey/unpublished data was sought No<br>1.6 The excluded studies are listed No<br>1.7 The relevant characteristics of the included studies are provided Yes<br>1.8 The scientific quality of the included studies was assessed and reported Yes<br>1.9 Was the scientific quality of the included studies used appropriately? Can't say<br>1.10 Appropriate methods are used to combine the individual study findings N/A<br>1.11 The likelihood of publication bias was assessed appropriately No	Low quality (−) - SIGN 1− Meta-analyses, systematic reviews of RCTs, or RCTs with a high risk of bias

Full Citation	Study Design	SIGN Tool	Item and response	Overall Risk of Bias / Confidence Rating
			1.12 Conflicts of interest are declared Yes	
9. Frank S, Capriotti J, Brown SM, Tessema B. Povidone-Iodine Use in Sinonasal and Oral Cavities: A Review of Safety in the COVID-19 Era. Ear Nose Throat J. 2020;99(9):586–93.	Narrative review	No SIGN methodology checklist applicable		
10. Gerges BZ, Rosenblatt J, Truong YL, Reitzel RA, Raad I. Review of allergic reactions from use of chlorhexidine on medical products in clinical settings over 40 years: Risks and mitigations. Infect Control Hosp Epidemiol. 2022;43(6):775–89.	Narrative review	No SIGN methodology checklist applicable		
11.Solderer A, Schmidlin PR. Literature review of aggregated evidence (umbrella review) on the allergy potential of chlorhexidine mouthrinse solutions. Quintessence Int. 2022;53(9):808–14.	Umbrella review / aggregated evidence review	Checklist 1: Systematic Reviews and Meta-Analyses	1.1 The research question is clearly defined and inclusion/exclusion criteria are listed Yes<br>1.2 A comprehensive literature search is carried out Yes<br>1.3 At least two people should have selected studies Yes<br>1.4 At least two people should have extracted data Yes<br>1.5 Grey/unpublished data was sought No<br>1.6 The excluded studies are listed No<br>1.7 The relevant characteristics of the included studies are provided Yes<br>1.8 The scientific quality of the included studies was assessed and reported Yes<br>1.9 Was the scientific quality of the included studies used appropriately? Can't say<br>1.10 Appropriate methods are used to combine the individual study findings N/A<br>1.11 The likelihood of publication bias was assessed appropriately No<br>1.12 Conflicts of interest are declared No	Low quality (−) - SIGN 1− Meta-analyses, systematic reviews of RCTs, or RCTs with a high risk of bias
12.do Amaral GCLS, Hassan MA, Sloniak MC, Pannuti CM, Romito GA, Villar CC. Effects of antimicrobial mouthwashes on the human oral microbiome: Systematic review of controlled clinical trials. Int J Dent Hyg. 2023;21(1):128–40.	Systematic review of controlled clinical trials	Checklist 1: Systematic Reviews and Meta-Analyses	1.1 The research question is clearly defined and inclusion/exclusion criteria are listed Yes<br>1.2 A comprehensive literature search is carried out Yes<br>1.3 At least two people should have selected studies Yes<br>1.4 At least two people should have extracted data Yes<br>1.5 Grey/unpublished data was sought Yes<br>1.6 The excluded studies are listed No	Acceptable (+) - SIGN 1+ Well-conducted meta-analyses, systematic reviews of RCTs, or RCTs

Full Citation	Study Design	SIGN Tool	Item and response	Overall Risk of Bias / Confidence Rating
			1.7 The relevant characteristics of the included studies are provided Yes<br>1.8 The scientific quality of the included studies was assessed and reported Yes<br>1.9 Was the scientific quality of the included studies used appropriately? Yes<br>1.10 Appropriate methods are used to combine the individual study findings N/A<br>1.11 The likelihood of publication bias was assessed appropriately No<br>1.12 Conflicts of interest are declared Yes	with a low risk of bias
13.Shamsher S, Suhaimi NHI, Noor Ali R, Zulkifli K. Combating chlorhexidine allergy in perioperative setting. Med J Malaysia. 2023;78(1):126–7.	Expert communication	No SIGN methodology checklist applicable		
**Block 2 · Preoperative preparation protocols for dentoalveolar surgery and implantology**				
14.Fotos PG, Koorbusch GF, Sarasin DS, Kist RJ. Evaluation of intra-alveolar chlorhexidine dressings after removal of impacted mandibular third molars. Oral Surg Oral Med Oral Pathol. 1992;73(3):383–8.	Non-randomised controlled clinical study	Checklist 5: Controlled Trials (Non-Randomised; items 1.2-1.4 omitted)	1.1 The study addresses an appropriate and clearly focused question Yes<br>1.5 The treatment and control groups are similar at the start of the trial Yes<br>1.6 The only difference between groups is the treatment under investigation Yes<br>1.7 All relevant outcomes are measured in a standard, valid and reliable way Yes<br>1.8 Percentage of individuals in each arm who dropped out before completion Not reported<br>1.9 All subjects analysed in the groups to which they were randomly allocated (ITT) Can't say<br>1.10 Where the study is carried out at more than one site, results are comparable for all sites Can't say	Low quality (−) - SIGN 2− Cohort or case-control studies with a high risk of bias
15.Hermesch CB, Hilton TJ, Biesbrock AR, Baker RA, Cain-Hamlin J, McClanahan SF, et al. Perioperative use of 0.12% chlorhexidine gluconate for the prevention of alveolar osteitis: efficacy and risk factor analysis. Oral Surg Oral Med Oral Pathol Oral Radiol Endod. 1998;85(4):381–7.	Prospective observational cohort	Checklist 3: Cohort Studies	1.1 The study addresses an appropriate and clearly focused question Yes<br>1.2 The two groups are selected from source populations comparable in all respects other than the factor under investigation No<br>1.3 The study indicates how many of the people asked to take part did so, in each group Can't say<br>1.4 Likelihood that eligible subjects might have the outcome at enrolment is assessed and taken into account No<br>1.5 Percentage of individuals recruited into each arm who dropped out before study completion Not reported	Low quality (−) - SIGN 2− Cohort or case-control studies with a high risk of bias

Full Citation	Study Design	SIGN Tool	Item and response	Overall Risk of Bias / Confidence Rating
			1.6 Comparison is made between full participants and those lost to follow up, by exposure status Can't say<br>1.7 The outcomes are clearly defined Can't say<br>1.8 The assessment of outcome is made blind to exposure status No<br>1.9 Where blinding was not possible, there is recognition that knowledge of exposure status could have influenced outcome assessment Yes<br>1.10 The method of assessment of exposure is reliable Yes<br>1.11 Evidence from other sources is used to demonstrate validity and reliability of outcome assessment No<br>1.12 Exposure level or prognostic factor is assessed more than once No<br>1.13 The main potential confounders are identified and taken into account in the design and analysis No<br>1.14 Have confidence intervals been provided? Can't say	
16.Kosutic D, Uglesic V, Perkovic D, Persic Z, Solman L, Lupi-Ferandin S, et al. Preoperative antiseptics in clean/contaminated maxillofacial and oral surgery: prospective randomized study. Int J Oral Maxillofac Surg. 2009;38(2):160–5.	Randomised controlled trial	Checklist 2: Controlled Trials	1.1 The study addresses an appropriate and clearly focused question Yes<br>1.2 The assignment of subjects to treatment groups is randomised Yes<br>1.3 An adequate concealment method is used Can't say<br>1.4 The design keeps subjects and investigators 'blind' about treatment allocation No<br>1.5 The treatment and control groups are similar at the start of the trial Yes<br>1.6 The only difference between groups is the treatment under investigation Yes<br>1.7 All relevant outcomes are measured in a standard, valid and reliable way Yes<br>1.8 Percentage of individuals in each arm who dropped out before completion Not reported<br>1.9 All subjects analysed in the groups to which they were randomly allocated (ITT) Can't say<br>1.10 Where the study is carried out at more than one site, results are comparable for all sites Yes	Acceptable (+) - SIGN 1+ Well-conducted meta-analyses, systematic reviews of RCTs, or RCTs with a low risk of bias
17.Torres-Lagares D, Gutierrez-Perez JL, Hita-Iglesias P, Magallanes-Abad N, Flores-Ruiz R, Basallote-Garcia M, et al. Randomized, double-blind study of effectiveness of intra-alveolar application of chlorhexidine gel in reducing incidence of alveolar	Randomised double-blind controlled trial	Checklist 2: Controlled Trials	1.1 The study addresses an appropriate and clearly focused question Yes<br>1.2 The assignment of subjects to treatment groups is randomised Yes<br>1.3 An adequate concealment method is used Yes<br>1.4 The design keeps subjects and investigators 'blind' about treatment allocation Yes<br>1.5 The treatment and control groups are similar at the start of the trial Yes<br>1.6 The only difference between groups is the treatment under investigation Yes	Acceptable (+) - SIGN 1+ Well-conducted meta-analyses, systematic reviews of RCTs, or RCTs with a low risk of bias

Full Citation	Study Design	SIGN Tool	Item and response	Overall Risk of Bias / Confidence Rating
osteitis and bleeding complications in mandibular third molar surgery in patients with bleeding disorders. J Oral Maxillofac Surg. 2010;68(6):1322–6.			1.7 All relevant outcomes are measured in a standard, valid and reliable way Yes<br>1.8 Percentage of individuals in each arm who dropped out before completion Not reported<br>1.9 All subjects analysed in the groups to which they were randomly allocated (ITT) Can't say<br>1.10 Where the study is carried out at more than one site, results are comparable for all sites Not applicable	
18.Babar A, Ibrahim MW, Baig NJ, Shah I, Amin E. Efficacy of intra-alveolar chlorhexidine gel in reducing frequency of alveolar osteitis in mandibular third molar surgery. J Coll Physicians Surg Pak. 2012;22(2):91–4.	Controlled clinical study	Checklist 5: Controlled Trials (Non-Randomised; items 1.2-1.4 omitted)	1.1 The study addresses an appropriate and clearly focused question Yes<br>1.5 The treatment and control groups are similar at the start of the trial No<br>1.6 The only difference between groups is the treatment under investigation No<br>1.7 All relevant outcomes are measured in a standard, valid and reliable way No<br>1.8 Percentage of individuals in each arm who dropped out before completion Not reported<br>1.9 All subjects analysed in the groups to which they were randomly allocated (ITT) No<br>1.10 Where the study is carried out at more than one site, results are comparable for all sites Not applicable	Low quality (−) - SIGN 2− Cohort or case-control studies with a high risk of bias
19.Maharaj B, Coovadia Y, Vayej AC. A comparative study of amoxicillin, clindamycin and chlorhexidine in the prevention of post-extraction bacteraemia. Cardiovasc J Afr. 2012;23(9):491–4.	Comparative study	Checklist 5: Controlled Trials (Non-Randomised; items 1.2-1.4 omitted)	1.1 The study addresses an appropriate and clearly focused question Yes<br>1.5 The treatment and control groups are similar at the start of the trial Yes<br>1.6 The only difference between groups is the treatment under investigation Yes<br>1.7 All relevant outcomes are measured in a standard, valid and reliable way Yes<br>1.8 Percentage of individuals in each arm who dropped out before completion Not reported<br>1.9 All subjects analysed in the groups to which they were randomly allocated (ITT) Yes<br>1.10 Where the study is carried out at more than one site, results are comparable for all sites Not applicable	Low quality (−) - SIGN 2− Cohort or case-control studies with a high risk of bias
20.Haraji A, Rakhshan V, Khamverdi N, Alishahi HK. Effects of intra-alveolar placement of 0.2% chlorhexidine bioadhesive gel on dry socket incidence and postsurgical	Randomised double-blind split-mouth controlled trial	Checklist 2: Controlled Trials	1.1 The study addresses an appropriate and clearly focused question Yes<br>1.2 The assignment of subjects to treatment groups is randomised Yes<br>1.3 An adequate concealment method is used Yes<br>1.4 The design keeps subjects and investigators 'blind' about treatment allocation Yes	High quality (++), SIGN 1++: randomized double-blind split-mouth

Full Citation	Study Design	SIGN Tool	Item and response	Overall Risk of Bias / Confidence Rating
pain: a double-blind split-mouth randomized controlled clinical trial. J Orofac Pain. 2013;27(3):256–62.			1.5 The treatment and control groups are similar at the start of the trial Yes<br>1.6 The only difference between groups is the treatment under investigation Yes<br>1.7 All relevant outcomes are measured in a standard, valid and reliable way Yes<br>1.8 Percentage of individuals in each arm who dropped out before completion Yes<br>1.9 All subjects analysed in the groups to which they were randomly allocated (ITT) Yes<br>1.10 Where the study is carried out at more than one site, results are comparable for all sites Not applicable	controlled clinical trial with very low risk of bias.
21.Haraji A, Rakhshan V. Chlorhexidine gel and less difficult surgeries might reduce post-operative pain, controlling for dry socket, infection and analgesic consumption: a split-mouth controlled randomised clinical trial. J Oral Rehabil. 2015;42(3):209–19.	Randomised split-mouth controlled trial	Checklist 2: Controlled Trials	1.1 The study addresses an appropriate and clearly focused question Yes<br>1.2 The assignment of subjects to treatment groups is randomised Yes<br>1.3 An adequate concealment method is used Yes<br>1.4 The design keeps subjects and investigators 'blind' about treatment allocation Yes<br>1.5 The treatment and control groups are similar at the start of the trial Yes<br>1.6 The only difference between groups is the treatment under investigation Yes<br>1.7 All relevant outcomes are measured in a standard, valid and reliable way Yes<br>1.8 Percentage of individuals in each arm who dropped out before completion Yes<br>1.9 All subjects analysed in the groups to which they were randomly allocated (ITT) Yes<br>1.10 Where the study is carried out at more than one site, results are comparable for all sites Not applicable	High quality (++), SIGN 1++: randomized split-mouth controlled clinical trial with very low risk of bias.
22.Requena-Calla S, Funes-Rumiche I. Effectiveness of intra-alveolar chlorhexidine gel in reducing dry socket following surgical extraction of lower third molars. A pilot study. J Clin Exp Dent. 2016;8(2):e160-163.	Pilot randomised controlled trial	Checklist 2: Controlled Trials	1.1 The study addresses an appropriate and clearly focused question Yes<br>1.2 The assignment of subjects to treatment groups is randomised Yes<br>1.3 An adequate concealment method is used Can't say<br>1.4 The design keeps subjects and investigators 'blind' about treatment allocation Yes<br>1.5 The treatment and control groups are similar at the start of the trial Yes<br>1.6 The only difference between groups is the treatment under investigation Can't say<br>1.7 All relevant outcomes are measured in a standard, valid and reliable way Yes	Low quality (−) – SIGN 1−. Randomised controlled trials with a high risk of bias.

Full Citation	Study Design	SIGN Tool	Item and response	Overall Risk of Bias / Confidence Rating
			1.8 Percentage of individuals in each arm who dropped out before completion Yes<br>1.9 All subjects analysed in the groups to which they were randomly allocated (ITT) Can't say<br>1.10 Where the study is carried out at more than one site, results are comparable for all sites Not applicable	
23.Kaur J, Raval R, Bansal A, Kumawat V. Repercussions of intraalveolar placement of combination of 0.2% chlorhexidine & 10 Mg metronidazole gel on the occurrence of dry sockets- A randomized control trial. J Clin Exp Dent. 2017;9(2):e284–8.	Randomised controlled trial	Checklist 2: Controlled Trials	1.1 The study addresses an appropriate and clearly focused question Yes<br>1.2 The assignment of subjects to treatment groups is randomised Yes<br>1.3 An adequate concealment method is used Yes<br>1.4 The design keeps subjects and investigators 'blind' about treatment allocation Yes<br>1.5 The treatment and control groups are similar at the start of the trial Yes<br>1.6 The only difference between groups is the treatment under investigation Yes<br>1.7 All relevant outcomes are measured in a standard, valid and reliable way Yes<br>1.8 Percentage of individuals in each arm who dropped out before completion Yes<br>1.9 All subjects analysed in the groups to which they were randomly allocated (ITT) Yes<br>1.10 Where the study is carried out at more than one site, results are comparable for all sites Not applicable	Acceptable (+) - SIGN 1+ Well-conducted meta-analyses, systematic reviews of RCTs, or RCTs with a low risk of bias
24.Coello-Gómez A, Navarro-Suárez S, Diosdado-Cano JM, Azcárate-Velazquez F, Bargiela-Pérez P, Serrera-Figallo MA, et al. Postoperative effects on lower third molars of using mouthwashes with super-oxidized solution versus 0.2% chlorhexidine gel: A randomized double-blind trial. Med Oral Patol Oral Cir Bucal. 2018;23(6):e716–22.	Randomised double-blind trial	Checklist 2: Controlled Trials	1.1 The study addresses an appropriate and clearly focused question Yes<br>1.2 The assignment of subjects to treatment groups is randomised Yes<br>1.3 An adequate concealment method is used Can't say<br>1.4 The design keeps subjects and investigators 'blind' about treatment allocation Yes<br>1.5 The treatment and control groups are similar at the start of the trial Yes<br>1.6 The only difference between groups is the treatment under investigation Yes<br>1.7 All relevant outcomes are measured in a standard, valid and reliable way Yes<br>1.8 Percentage of individuals in each arm who dropped out before completion Yes<br>1.9 All subjects analysed in the groups to which they were randomly allocated (ITT) Yes	Acceptable (+) - SIGN 1+ Well-conducted meta-analyses, systematic reviews of RCTs, or RCTs with a low risk of bias

Full Citation	Study Design	SIGN Tool	Item and response	Overall Risk of Bias / Confidence Rating
			1.10 Where the study is carried out at more than one site, results are comparable for all sites Not applicable	
25.Jadhao VA, Rao A, Hande P, Mahajani M, Raktade PP, Gedam R, et al. The Efficiency of Three Irrigating Solutions after Surgical Removal of Impacted Mandibular Third Molars: A Cross-sectional Study. J Contemp Dent Pract. 2018;19(9):1147–51.	Cross-sectional comparative study	Checklist 5: Controlled Trials (Non-Randomised; items 1.2-1.4 omitted)	1.1 The study addresses an appropriate and clearly focused question Yes<br>1.5 The treatment and control groups are similar at the start of the trial Can't say<br>1.6 The only difference between groups is the treatment under investigation No<br>1.7 All relevant outcomes are measured in a standard, valid and reliable way Can't say<br>1.8 Percentage of individuals in each arm who dropped out before completion Yes<br>1.9 All subjects analysed in the groups to which they were randomly allocated (ITT) N/A<br>1.10 Where the study is carried out at more than one site, results are comparable for all sites Not applicable	Low quality (–), SIGN 2−: cross-sectional comparative study with high risk of bias and inappropriate design for assessing treatment effectiveness.
26.Muñoz-Cámara D, Pardo-Zamora G, Camacho-Alonso F. Postoperative effects of intra-alveolar application of 0.2% chlorhexidine or 1% hyaluronic acid bioadhesive gels after mandibular third molar extraction: a double-blind randomized controlled clinical trial. Clin Oral Investig. 2021;25(2):617–25.	Randomised double-blind controlled clinical trial	Checklist 2: Controlled Trials	1.1 The study addresses an appropriate and clearly focused question Yes<br>1.2 The assignment of subjects to treatment groups is randomised Yes<br>1.3 An adequate concealment method is used Yes<br>1.4 The design keeps subjects and investigators 'blind' about treatment allocation Yes<br>1.5 The treatment and control groups are similar at the start of the trial Yes<br>1.6 The only difference between groups is the treatment under investigation Yes<br>1.7 All relevant outcomes are measured in a standard, valid and reliable way Yes<br>1.8 Percentage of individuals in each arm who dropped out before completion Yes<br>1.9 All subjects analysed in the groups to which they were randomly allocated (ITT) Yes<br>1.10 Where the study is carried out at more than one site, results are comparable for all sites Not applicable	High quality (++), SIGN 1++: randomized double-blind controlled clinical trial with very low risk of bias.
27.Dorestan N, Ostadian N, Mahmoudinezhad SS, Farajzadeh Sheikh A, Moradi K. Comparative efficacy of 0.2% chlorhexidine and	Randomised clinical trial	Checklist 2: Controlled Trials	1.1 The study addresses an appropriate and clearly focused question Yes<br>1.2 The assignment of subjects to treatment groups is randomised No<br>1.3 An adequate concealment method is used No	Acceptable (+) - SIGN 2+ Well-conducted non-randomised

Full Citation	Study Design	SIGN Tool	Item and response	Overall Risk of Bias / Confidence Rating
10% Betadine for perioral skin disinfection prior to oral surgical procedures: a clinical trial. Ir J Med Sci. 2022;191(5):2207–11.			1.4 The design keeps subjects and investigators 'blind' about treatment allocation No<br>1.5 The treatment and control groups are similar at the start of the trial Yes<br>1.6 The only difference between groups is the treatment under investigation Yes<br>1.7 All relevant outcomes are measured in a standard, valid and reliable way Yes<br>1.8 Percentage of individuals in each arm who dropped out before completion Yes<br>1.9 All subjects analysed in the groups to which they were randomly allocated (ITT) Yes<br>1.10 Where the study is carried out at more than one site, results are comparable for all sites Not applicable	comparative study with a low to moderate risk of bias.
28.Sruthy TV, Roshni A, Aslam S, Cherian MP, Soman S, Akhila K. Low-concentration Povidone-iodine and Normal Saline as Irrigant on Reducing Postoperative Complications after the Third-molar Surgery: A Comparative Study. J Contemp Dent Pract. 2022;23(7):733–8.	Non-randomised comparative study	Checklist 5: Controlled Trials (Non-Randomised; items 1.2-1.4 omitted)	1.1 The study addresses an appropriate and clearly focused question Yes<br>1.5 The treatment and control groups are similar at the start of the trial Yes<br>1.6 The only difference between groups is the treatment under investigation Yes<br>1.7 All relevant outcomes are measured in a standard, valid and reliable way Can't say<br>1.8 Percentage of individuals in each arm who dropped out before completion Yes<br>1.9 All subjects analysed in the groups to which they were randomly allocated (ITT) Yes<br>1.10 Where the study is carried out at more than one site, results are comparable for all sites Not applicable	Low quality (−) - SIGN 2− Cohort or case-control studies with a high risk of bias
29.Vijayakumar G, Sundaram GA, Kumar SP, Krishna VK, Krishnan M. Comparison of the Effectiveness of Four Different Irrigation Solutions on Postoperative Sequelae in Patients Undergoing Lower Third Molar Surgery: A Prospective Study. Cureus. 2023;15(12):e50816.	Non-randomised prospective observational study	Checklist 5: Controlled Trials (Non-Randomised; items 1.2-1.4 omitted)	1.1 The study addresses an appropriate and clearly focused question Yes<br>1.5 The treatment and control groups are similar at the start of the trial Yes<br>1.6 The only difference between groups is the treatment under investigation Yes<br>1.7 All relevant outcomes are measured in a standard, valid and reliable way Can't say<br>1.8 Percentage of individuals in each arm who dropped out before completion Can't say<br>1.9 All subjects analysed in the groups to which they were randomly allocated (ITT) Yes<br>1.10 Where the study is carried out at more than one site, results are comparable for all sites Not applicable	Low quality (−) - SIGN 2− Cohort or case-control studies with a high risk of bias

Full Citation	Study Design	SIGN Tool	Item and response	Overall Risk of Bias / Confidence Rating
30.Weber J, Bonn EL, Auer DL, Kirschneck C, Buchalla W, Scholz KJ, et al. Preprocedural mouthwashes for infection control in dentistry-an update. Clin Oral Investig. 2023;27(Suppl 1):33–44.	Narrative review	No SIGN methodology checklist applicable		
31.Gumrukcu Z, Karabag M, Guven SE. The effects of chlorhexidine gel and tranexamic acid application after tooth extraction on the risk of alveolar osteitis formation: a double blind clinical study. Clin Oral Investig. 2024;28(9):494.	Randomised double-blind clinical study	Checklist 2: Controlled Trials	1.1 The study addresses an appropriate and clearly focused question Yes<br>1.2 The assignment of subjects to treatment groups is randomised Yes<br>1.3 An adequate concealment method is used Can't say<br>1.4 The design keeps subjects and investigators 'blind' about treatment allocation Yes<br>1.5 The treatment and control groups are similar at the start of the trial Yes<br>1.6 The only difference between groups is the treatment under investigation Yes<br>1.7 All relevant outcomes are measured in a standard, valid and reliable way Yes<br>1.8 Percentage of individuals in each arm who dropped out before completion Yes<br>1.9 All subjects analysed in the groups to which they were randomly allocated (ITT) Yes<br>1.10 Where the study is carried out at more than one site, results are comparable for all sites Not applicable	Acceptable (+) - SIGN 1+ Well-conducted meta-analyses, systematic reviews of RCTs, or RCTs with a low risk of bias
32.Shahraki M, Khazaei AH, Hakimi S, Hasheminasab M, Haradasht SA. Effect of chlorhexidine, povidone-iodine, and hydrogen peroxide irrigation on pain and swelling after mandibular third molar surgery: randomized controlled trial. Int J Oral Maxillofac Surg. 2025;54(7):677–83.	Randomised controlled trial	Checklist 2: Controlled Trials	1.1 The study addresses an appropriate and clearly focused question Yes<br>1.2 The assignment of subjects to treatment groups is randomised Yes<br>1.3 An adequate concealment method is used Yes<br>1.4 The design keeps subjects and investigators 'blind' about treatment allocation Can't say<br>1.5 The treatment and control groups are similar at the start of the trial Yes<br>1.6 The only difference between groups is the treatment under investigation Can't say<br>1.7 All relevant outcomes are measured in a standard, valid and reliable way Yes<br>1.8 Percentage of individuals in each arm who dropped out before completion Yes<br>1.9 All subjects analysed in the groups to which they were randomly allocated (ITT) Yes	Acceptable (+) - SIGN 1+ Well-conducted meta-analyses, systematic reviews of RCTs, or RCTs with a low risk of bias

Full Citation	Study Design	SIGN Tool	Item and response	Overall Risk of Bias / Confidence Rating
			1.10 Where the study is carried out at more than one site, results are comparable for all sites Not applicable	
**Block 3 · Postoperative Care Protocols**				
33.Sweet JB, Macynski AA. Effect of antimicrobial mouth rinses on the incidence of localized alveolitis and infection following mandibular third molar oral surgery. Oral Surg Oral Med Oral Pathol. 1985;59(1):24–6.	Retrospective controlled study	Checklist 5: Controlled Trials (Non-Randomised; items 1.2-1.4 omitted)	1.1 The study addresses an appropriate and clearly focused question Yes<br>1.5 The treatment and control groups are similar at the start of the trial Can't say<br>1.6 The only difference between groups is the treatment under investigation Can't say<br>1.7 All relevant outcomes are measured in a standard, valid and reliable way Yes<br>1.8 Percentage of individuals in each arm who dropped out before completion Not reported<br>1.9 All subjects analysed in the groups to which they were randomly allocated (ITT) N/A<br>1.10 Where the study is carried out at more than one site, results are comparable for all sites Can't say	Unacceptable (0) - critical risk of bias - SIGN 2− Cohort or case-control studies with a high risk of bias
34.Lambert PM, Morris HF, Ochi S. The influence of 0.12% chlorhexidine digluconate rinses on the incidence of infectious complications and implant success. J Oral Maxillofac Surg. 1997;55(12 Suppl 5):25–30.	Prospective observational study	Checklist 3: Cohort Studies	1.1 The study addresses an appropriate and clearly focused question Yes<br>1.2 The two groups are selected from source populations comparable in all respects other than the factor under investigation Can't say<br>1.3 The study indicates how many of the people asked to take part did so, in each group Can't say<br>1.4 Likelihood that eligible subjects might have the outcome at enrolment is assessed and taken into account No<br>1.5 Percentage of individuals recruited into each arm who dropped out before study completion Not reported<br>1.6 Comparison is made between full participants and those lost to follow up, by exposure status Can't say<br>1.7 The outcomes are clearly defined Yes<br>1.8 The assessment of outcome is made blind to exposure status No<br>1.9 Where blinding was not possible, there is recognition that knowledge of exposure status could have influenced outcome assessment Yes<br>1.10 The method of assessment of exposure is reliable Yes<br>1.11 Evidence from other sources is used to demonstrate validity and reliability of outcome assessment No<br>1.12 Exposure level or prognostic factor is assessed more than once No<br>1.13 The main potential confounders are identified and taken into account in the design and analysis No<br>1.14 Have confidence intervals been provided? No	Low quality (−) - SIGN 2− Cohort or case-control studies with a high risk of bias

Full Citation	Study Design	SIGN Tool	Item and response	Overall Risk of Bias / Confidence Rating
35.Mahmoud Hashemi H, Mohammadi F, Hasheminasab M, Mahmoud Hashemi A, Zahraei S, Mahmoud Hashemi T. Effect of low-concentration povidone iodine on postoperative complications after third molar surgery: a pilot split-mouth study. J Oral Maxillofac Surg. 2015;73(1):18–21.	Pilot split-mouth randomised controlled study	Checklist 2: Controlled Trials	1.1 The study addresses an appropriate and clearly focused question Yes<br>1.2 The assignment of subjects to treatment groups is randomised Yes<br>1.3 An adequate concealment method is used Yes<br>1.4 The design keeps subjects and investigators 'blind' about treatment allocation No<br>1.5 The treatment and control groups are similar at the start of the trial Yes<br>1.6 The only difference between groups is the treatment under investigation Yes<br>1.7 All relevant outcomes are measured in a standard, valid and reliable way Yes<br>1.8 Percentage of individuals in each arm who dropped out before completion Not reported<br>1.9 All subjects analysed in the groups to which they were randomly allocated (ITT) Yes<br>1.10 Where the study is carried out at more than one site, results are comparable for all sites N/A	Acceptable (+) - SIGN 1+ Well-conducted meta-analyses, systematic reviews of RCTs, or RCTs with a low risk of bias
36.Lopez-Lopez J, Jan-Pallí E, lez-Navarro BG, Jané-Salas E, Estrugo-Devesa A, Milani M. Efficacy of chlorhexidine, dexpanthenol, allantoin and chitosan gel in comparison with bicarbonate oral rinse in controlling post-interventional inflammation, pain and cicatrization in subjects undergoing dental surgery. Curr Med Res Opin. 2015;31(12):2179–83.	Randomised comparative clinical trial	Checklist 2: Controlled Trials	1.1 The study addresses an appropriate and clearly focused question Yes<br>1.2 The assignment of subjects to treatment groups is randomised Yes<br>1.3 An adequate concealment method is used Can’t say<br>1.4 The design keeps subjects and investigators 'blind' about treatment allocation Can't say<br>1.5 The treatment and control groups are similar at the start of the trial Yes<br>1.6 The only difference between groups is the treatment under investigation Yes<br>1.7 All relevant outcomes are measured in a standard, valid and reliable way Yes<br>1.8 Percentage of individuals in each arm who dropped out before completion Not reported<br>1.9 All subjects analysed in the groups to which they were randomly allocated (ITT) Can't say<br>1.10 Where the study is carried out at more than one site, results are comparable for all sites N/A	Acceptable (+) - SIGN 1+ Well-conducted meta-analyses, systematic reviews of RCTs, or RCTs with a low risk of bias

Full Citation	Study Design	SIGN Tool	Item and response	Overall Risk of Bias / Confidence Rating
37.Madrazo-Jiménez M, Rodríguez-Caballero Á, Serrera-Figallo MÁ, Garrido-Serrano R, Gutiérrez-Corrales A, Gutiérrez-Pérez JL, et al. The effects of a topical gel containing chitosan, 0,2% chlorhexidine, allantoin and despanthenol on the wound healing process subsequent to impacted lower third molar extraction. Med Oral Patol Oral Cir Bucal. 2016;21(6):e696–702.	Non-randomised controlled clinical study	Checklist 5: Controlled Trials (Non-Randomised; items 1.2-1.4 omitted)	1.1 The study addresses an appropriate and clearly focused question Yes<br>1.5 The treatment and control groups are similar at the start of the trial Yes<br>1.6 The only difference between groups is the treatment under investigation Yes<br>1.7 All relevant outcomes are measured in a standard, valid and reliable way Yes<br>1.8 Percentage of individuals in each arm who dropped out before completion Not reported<br>1.9 All subjects analysed in the groups to which they were randomly allocated (ITT) Can't say<br>1.10 Where the study is carried out at more than one site, results are comparable for all sites N/A	Low quality (−) - SIGN 2− Cohort or case-control studies with a high risk of bias
38.Cho H, Lynham AJ, Hsu E. Postoperative interventions to reduce inflammatory complications after third molar surgery: review of the current evidence. Aust Dent J. 2017;62(4):412–9.	Narrative review	No SIGN methodology checklist applicable		
39.Osunde OD, Anyanechi CE, Bassey GO. Prevention of alveolar osteitis after third molar surgery: Comparative study of the effect of warm saline and chlorhexidine mouth rinses. Niger J Clin Pract. 2017;20(4):470–3.	Non-randomised comparative clinical study	Checklist 5: Controlled Trials (Non-Randomised; items 1.2-1.4 omitted)	1.1 The study addresses an appropriate and clearly focused question Yes<br>1.5 The treatment and control groups are similar at the start of the trial Yes<br>1.6 The only difference between groups is the treatment under investigation Yes<br>1.7 All relevant outcomes are measured in a standard, valid and reliable way Yes<br>1.8 Percentage of individuals in each arm who dropped out before completion N/A<br>1.9 All subjects analysed in the groups to which they were randomly allocated (ITT) Yes<br>1.10 Where the study is carried out at more than one site, results are comparable for all sites N/A	Low quality (−) - SIGN 2− Cohort or case-control studies with a high risk of bias

Full Citation	Study Design	SIGN Tool	Item and response	Overall Risk of Bias / Confidence Rating
40.Teshome A. The efficacy of chlorhexidine gel in the prevention of alveolar osteitis after mandibular third molar extraction: a systematic review and meta-analysis. BMC Oral Health. 2017;17(1):82.	Systematic review and meta-analysis	Checklist 1: Systematic Reviews and Meta-Analyses	1.1 The research question is clearly defined and inclusion/exclusion criteria are listed Yes<br>1.2 A comprehensive literature search is carried out Yes<br>1.3 At least two people should have selected studies yES<br>1.4 At least two people should have extracted data Can't say<br>1.5 Grey/unpublished data was sought No<br>1.6 The excluded studies are listed No<br>1.7 The relevant characteristics of the included studies are provided Yes<br>1.8 The scientific quality of the included studies was assessed and reported Yes<br>1.9 Was the scientific quality of the included studies used appropriately? Yes<br>1.10 Appropriate methods are used to combine the individual study findings Yes<br>1.11 The likelihood of publication bias was assessed appropriately Yes<br>1.12 Conflicts of interest are declared Yes	Acceptable (+) - SIGN 1+ Well-conducted meta-analyses, systematic reviews of RCTs, or RCTs with a low risk of bias
41.Coello-Gómez A, Navarro-Suárez S, Diosdado-Cano JM, Azcárate-Velazquez F, Bargiela-Pérez P, Serrera-Figallo MA, et al. Postoperative effects on lower third molars of using mouthwashes with super-oxidized solution versus 0.2% chlorhexidine gel: A randomized double-blind trial. Med Oral Patol Oral Cir Bucal. 2018;23(6):e716–22.	Randomised double-blind trial	Checklist 2: Controlled Trials	1.1 The study addresses an appropriate and clearly focused question Yes<br>1.2 The assignment of subjects to treatment groups is randomised Yes<br>1.3 An adequate concealment method is used Can't say<br>1.4 The design keeps subjects and investigators 'blind' about treatment allocation Yes<br>1.5 The treatment and control groups are similar at the start of the trial Yes<br>1.6 The only difference between groups is the treatment under investigation Yes<br>1.7 All relevant outcomes are measured in a standard, valid and reliable way Yes<br>1.8 Percentage of individuals in each arm who dropped out before completion No<br>1.9 All subjects analysed in the groups to which they were randomly allocated (ITT) Yes<br>1.10 Where the study is carried out at more than one site, results are comparable for all sites N/A	Acceptable (+) - SIGN 1+ Well-conducted meta-analyses, systematic reviews of RCTs, or RCTs with a low risk of bias
42.Amora-Silva BF, Ribeiro SC, Vieira CL, Mendes FR, Vieira-Neto AE, Abdon APV, et al. Clinical efficacy of new α-bisabolol mouthwashes in postoperative	Randomised controlled triple-blind clinical trial	Checklist 2: Controlled Trials	1.1 The study addresses an appropriate and clearly focused question Yes<br>1.2 The assignment of subjects to treatment groups is randomised Yes<br>1.3 An adequate concealment method is used Yes<br>1.4 The design keeps subjects and investigators 'blind' about treatment allocation Yes	High quality (++) - SIGN 1++ High quality meta-analyses, systematic reviews

Full Citation	Study Design	SIGN Tool	Item and response	Overall Risk of Bias / Confidence Rating
complications of maxillofacial surgeries: a randomized, controlled, triple-blind clinical trial. Clin Oral Investig. 2019;23(2):577–84.			1.5 The treatment and control groups are similar at the start of the trial Can't say<br>1.6 The only difference between groups is the treatment under investigation Can't say<br>1.7 All relevant outcomes are measured in a standard, valid and reliable way Yes<br>1.8 Percentage of individuals in each arm who dropped out before completion Not reported<br>1.9 All subjects analysed in the groups to which they were randomly allocated (ITT) Can't say<br>1.10 Where the study is carried out at more than one site, results are comparable for all sites N/A	of RCTs, or RCTs with a very low risk of bias
43.Halabi D, Escobar J, Alvarado C, Martinez N, Muñoz C. Chlorhexidine for prevention of alveolar osteitis: a randomised clinical trial. J Appl Oral Sci. 2018;26:e20170245.	Randomised clinical trial	Checklist 2: Controlled Trials	1.1 The study addresses an appropriate and clearly focused question Yes<br>1.2 The assignment of subjects to treatment groups is randomised Yes<br>1.3 An adequate concealment method is used Yes<br>1.4 The design keeps subjects and investigators 'blind' about treatment allocation Can't say<br>1.5 The treatment and control groups are similar at the start of the trial Yes<br>1.6 The only difference between groups is the treatment under investigation Yes<br>1.7 All relevant outcomes are measured in a standard, valid and reliable way Yes<br>1.8 Percentage of individuals in each arm who dropped out before completion 0%<br>1.9 All subjects analysed in the groups to which they were randomly allocated (ITT) Yes<br>1.10 Where the study is carried out at more than one site, results are comparable for all sites N/A	Acceptable (+) - SIGN 1+ Well-conducted meta-analyses, systematic reviews of RCTs, or RCTs with a low risk of bias
44.Solderer A, Kaufmann M, Hofer D, Wiedemeier D, Attin T, Schmidlin PR. Efficacy of chlorhexidine rinses after periodontal or implant surgery: a systematic review. Clin Oral Investig. 2019;23(1):21–32.	Systematic review	Checklist 1: Systematic Reviews and Meta-Analyses	1.1 The research question is clearly defined and inclusion/exclusion criteria are listed Yes<br>1.2 A comprehensive literature search is carried out Yes<br>1.3 At least two people should have selected studies Yes<br>1.4 At least two people should have extracted data Yes<br>1.5 Grey/unpublished data was sought No<br>1.6 The excluded studies are listed No<br>1.7 The relevant characteristics of the included studies are provided Yes<br>1.8 The scientific quality of the included studies was assessed and reported Yes	Acceptable (+) - SIGN 1+ Well-conducted meta-analyses, systematic reviews of RCTs, or RCTs with a low risk of bias

Full Citation	Study Design	SIGN Tool	Item and response	Overall Risk of Bias / Confidence Rating
			1.9 Was the scientific quality of the included studies used appropriately? Yes<br>1.10 Appropriate methods are used to combine the individual study findings N/A<br>1.11 The likelihood of publication bias was assessed appropriately No<br>1.12 Conflicts of interest are declared Yes	
45.Canellas JVDS, Fraga SRG, Santoro MF, Netto J de NS, Tinoco EMB. Intrasocket interventions to prevent alveolar osteitis after mandibular third molar surgery: A systematic review and network meta-analysis. J Craniomaxillofac Surg. 2020;48(9):902–13.	Systematic review and network meta-analysis	Checklist 1: Systematic Reviews and Meta-Analyses	1.1 The research question is clearly defined and inclusion/exclusion criteria are listed Yes<br>1.2 A comprehensive literature search is carried out Yes<br>1.3 At least two people should have selected studies Yes<br>1.4 At least two people should have extracted data Yes<br>1.5 Grey/unpublished data was sought Yes<br>1.6 The excluded studies are listed Yes<br>1.7 The relevant characteristics of the included studies are provided Yes<br>1.8 The scientific quality of the included studies was assessed and reported Yes<br>1.9 Was the scientific quality of the included studies used appropriately? Yes<br>1.10 Appropriate methods are used to combine the individual study findings Yes<br>1.11 The likelihood of publication bias was assessed appropriately No<br>1.12 Conflicts of interest are declared Yes	Acceptable (+) - SIGN 1+ Well-conducted meta-analyses, systematic reviews of RCTs, or RCTs with a low risk of bias
46.Sáez-Alcaide LM, Molinero-Mourelle P, González-Serrano J, Rubio-Alonso L, Bornstein MM, López-Quiles J. Efficacy of a topical gel containing chitosan, chlorhexidine, allantoin and dexpanthenol for pain and inflammation control after third molar surgery: A randomized and placebo-controlled clinical trial. Med Oral Patol Oral Cir Bucal. 2020;25(5):e644–51.	Randomised placebo-controlled clinical trial	Checklist 2: Controlled Trials	1.1 The study addresses an appropriate and clearly focused question Yes<br>1.2 The assignment of subjects to treatment groups is randomised Yes<br>1.3 An adequate concealment method is used Can't say<br>1.4 The design keeps subjects and investigators 'blind' about treatment allocation Yes<br>1.5 The treatment and control groups are similar at the start of the trial Yes<br>1.6 The only difference between groups is the treatment under investigation Yes<br>1.7 All relevant outcomes are measured in a standard, valid and reliable way Yes<br>1.8 Percentage of individuals in each arm who dropped out before completion 5%<br>1.9 All subjects analysed in the groups to which they were randomly allocated (ITT) Yes<br>1.10 Where the study is carried out at more than one site, results are comparable for all sites N/A	Acceptable (+) - SIGN 1+ Well-conducted meta-analyses, systematic reviews of RCTs, or RCTs with a low risk of bias

**Full Citation**	**Study Design**	**SIGN Tool**	**Item and response**	**Overall Risk of Bias / Confidence Rating**
47.Kaplan V, Hasanoglu Erbasar GN, Cigerim L, Altay Turgut H, Cerit A. Effect of St. John's wort oil and olive oil on the postoperative complications after third molar surgery: randomized, double-blind clinical trial. Clin Oral Investig. 2021;25(4):2429–38.	Randomised double-blind clinical trial	Checklist 2: Controlled Trials	1.1 The study addresses an appropriate and clearly focused question Yes<br>1.2 The assignment of subjects to treatment groups is randomised Yes<br>1.3 An adequate concealment method is used Can't say<br>1.4 The design keeps subjects and investigators 'blind' about treatment allocation Yes<br>1.5 The treatment and control groups are similar at the start of the trial Yes<br>1.6 The only difference between groups is the treatment under investigation Yes<br>1.7 All relevant outcomes are measured in a standard, valid and reliable way Yes<br>1.8 Percentage of individuals in each arm who dropped out before completion 0%<br>1.9 All subjects analysed in the groups to which they were randomly allocated (ITT) Yes<br>1.10 Where the study is carried out at more than one site, results are comparable for all sites N/A	Acceptable (+) - SIGN 1+ Well-conducted meta-analyses, systematic reviews of RCTs, or RCTs with a low risk of bias
48.Mohanty R, Jha C. Randomized Study on Postoperative Intra-alveolar Betadine Irrigation Versus Chlorhexidine Irrigation on the Incidence of Occurrence of Alveolar Osteitis After Mandibular Third Molar Surgery. J Maxillofac Oral Surg. 2022;21(1):163–7.	Randomised study	Checklist 2: Controlled Trials	1.1 The study addresses an appropriate and clearly focused question Yes<br>1.2 The assignment of subjects to treatment groups is randomised Can't say<br>1.3 An adequate concealment method is used No<br>1.4 The design keeps subjects and investigators 'blind' about treatment allocation No<br>1.5 The treatment and control groups are similar at the start of the trial Can't say<br>1.6 The only difference between groups is the treatment under investigation Can't say<br>1.7 All relevant outcomes are measured in a standard, valid and reliable way Yes<br>1.8 Percentage of individuals in each arm who dropped out before completion 5%<br>1.9 All subjects analysed in the groups to which they were randomly allocated (ITT) Can't say<br>1.10 Where the study is carried out at more than one site, results are comparable for all sites N/A	Acceptable (+) - SIGN 1+ Well-conducted meta-analyses, systematic reviews of RCTs, or RCTs with a low risk of bias
49.Cao Y, Jiang Q, Hu J. Prophylactic therapy for prevention of surgical site infection after extraction of third molar: An	Overview of systematic reviews	Checklist 1: Systematic Reviews and Meta-Analyses	1.1 The research question is clearly defined and inclusion/exclusion criteria are listed Yes<br>1.2 A comprehensive literature search is carried out Yes<br>1.3 At least two people should have selected studies Yes<br>1.4 At least two people should have extracted data Yes	Acceptable (+) - SIGN 1+ Well-conducted meta-analyses, systematic reviews

Full Citation	Study Design	SIGN Tool	Item and response	Overall Risk of Bias / Confidence Rating
overview of reviews. Med Oral Patol Oral Cir Bucal. 2023;28(6):e581–7.			1.5 Grey/unpublished data was sought No<br>1.6 The excluded studies are listed No<br>1.7 The relevant characteristics of the included studies are provided Yes<br>1.8 The scientific quality of the included studies was assessed and reported Yes<br>1.9 Was the scientific quality of the included studies used appropriately? Yes<br>1.10 Appropriate methods are used to combine the individual study findings N/A<br>1.11 The likelihood of publication bias was assessed appropriately No<br>1.12 Conflicts of interest are declared Yes	of RCTs, or RCTs with a low risk of bias
50.Erbasar GNH, Kaplan V, Cigerim L, Konarili FN, Sahin M. Effect of combined boric acid and chlorhexidine mouthwashes on postoperative complications and periodontal healing after impacted third molar surgery: a-double blind randomized study. Clin Oral Investig. 2023;27(7):3817–26.	Randomised double-blind study	Checklist 2: Controlled Trials	1.1 The study addresses an appropriate and clearly focused question Yes<br>1.2 The assignment of subjects to treatment groups is randomised Yes<br>1.3 An adequate concealment method is used Yes<br>1.4 The design keeps subjects and investigators 'blind' about treatment allocation Yes<br>1.5 The treatment and control groups are similar at the start of the trial Yes<br>1.6 The only difference between groups is the treatment under investigation Yes<br>1.7 All relevant outcomes are measured in a standard, valid and reliable way Yes<br>1.8 Percentage of individuals in each arm who dropped out before completion 0%<br>1.9 All subjects analysed in the groups to which they were randomly allocated (ITT) Yes<br>1.10 Where the study is carried out at more than one site, results are comparable for all sites N/A	Acceptable (+) - SIGN 1+ Well-conducted meta-analyses, systematic reviews of RCTs, or RCTs with a low risk of bias
51.Ajmera S, Sharma S, Sharma AK, Sharma V, Petchiammal SM, Sinha S. Aloe vera vs chlorhexidine in preventing alveolar osteitis. Bioinformation. 2024;20(9):993–7.	Randomised clinical study	Checklist 2: Controlled Trials	1.1 The study addresses an appropriate and clearly focused question Yes<br>1.2 The assignment of subjects to treatment groups is randomised No<br>1.3 An adequate concealment method is used No<br>1.4 The design keeps subjects and investigators 'blind' about treatment allocation No<br>1.5 The treatment and control groups are similar at the start of the trial Can't say<br>1.6 The only difference between groups is the treatment under investigation Can't say<br>1.7 All relevant outcomes are measured in a standard, valid and reliable way Can't say	Acceptable (+) - SIGN 1+ Well-conducted meta-analyses, systematic reviews of RCTs, or RCTs with a low risk of bias

Full Citation	Study Design	SIGN Tool	Item and response	Overall Risk of Bias / Confidence Rating
			1.8 Percentage of individuals in each arm who dropped out before completion Not reported<br>1.9 All subjects analysed in the groups to which they were randomly allocated (ITT) Can't say<br>1.10 Where the study is carried out at more than one site, results are comparable for all sites Can't say	
52.Boccalari E, Khijmatgar S, Occhipinti C, Del Fabbro M, Inchingolo F, Tartaglia GM. Effect of hydrogen peroxide and hyaluronic acid in mouth rinse after third molar extraction: a triple-blind parallel randomized controlled clinical trial. Eur Rev Med Pharmacol Sci. 2024;28(13):3946–57.	Randomised triple-blind parallel controlled clinical trial	Checklist 2: Controlled Trials	1.1 The study addresses an appropriate and clearly focused question Yes<br>1.2 The assignment of subjects to treatment groups is randomised Yes<br>1.3 An adequate concealment method is used Yes<br>1.4 The design keeps subjects and investigators 'blind' about treatment allocation Yes<br>1.5 The treatment and control groups are similar at the start of the trial Yes<br>1.6 The only difference between groups is the treatment under investigation Yes<br>1.7 All relevant outcomes are measured in a standard, valid and reliable way Yes<br>1.8 Percentage of individuals in each arm who dropped out before completion 0%<br>1.9 All subjects analysed in the groups to which they were randomly allocated (ITT) Yes<br>1.10 Where the study is carried out at more than one site, results are comparable for all sites N/A	High quality (++) - SIGN 1++ High quality meta-analyses, systematic reviews of RCTs, or RCTs with a very low risk of bias
53.Chugh A, P G G, Kumar P, Kaur A. Localised 0.2% chlorhexidine irrigation delivery system versus oral antibiotics in reducing postoperative complications in the surgical extraction of impacted mandibular third molar (IMTM). - a randomised controlled trial. Med Oral Patol Oral Cir Bucal. 2024;29(5):e690–7.	Randomised controlled trial	Checklist 2: Controlled Trials	1.1 The study addresses an appropriate and clearly focused question Yes<br>1.2 The assignment of subjects to treatment groups is randomised Yes<br>1.3 An adequate concealment method is used Yes<br>1.4 The design keeps subjects and investigators 'blind' about treatment allocation Yes<br>1.5 The treatment and control groups are similar at the start of the trial Yes<br>1.6 The only difference between groups is the treatment under investigation Yes<br>1.7 All relevant outcomes are measured in a standard, valid and reliable way Yes<br>1.8 Percentage of individuals in each arm who dropped out before completion 0%<br>1.9 All subjects analysed in the groups to which they were randomly allocated (ITT) Yes	Acceptable (+) - SIGN 1+ Well-conducted meta-analyses, systematic reviews of RCTs, or RCTs with a low risk of bias

Full Citation	Study Design	SIGN Tool	Item and response	Overall Risk of Bias / Confidence Rating
			1.10 Where the study is carried out at more than one site, results are comparable for all sites N/A	
54.Sakka S, Kharma MY, Al Rafedah A. Chlorhexidine mouthwash and augmentin to prevent Alveolar Osteitis after removal of mandibular third molar: a three-arm placebo- controlled randomized clinical trial. Afr Health Sci. 2024;24(2):117–23.	Three-arm placebo-controlled randomised clinical trial	Checklist 2: Controlled Trials	1.1 The study addresses an appropriate and clearly focused question Yes<br>1.2 The assignment of subjects to treatment groups is randomised Yes<br>1.3 An adequate concealment method is used Can't say<br>1.4 The design keeps subjects and investigators 'blind' about treatment allocation No<br>1.5 The treatment and control groups are similar at the start of the trial Can't say<br>1.6 The only difference between groups is the treatment under investigation Can't say<br>1.7 All relevant outcomes are measured in a standard, valid and reliable way Yes<br>1.8 Percentage of individuals in each arm who dropped out before completion 1%<br>1.9 All subjects analysed in the groups to which they were randomly allocated (ITT) Yes<br>1.10 Where the study is carried out at more than one site, results are comparable for all sites N/A	Acceptable (+) - SIGN 1+ Well-conducted meta-analyses, systematic reviews of RCTs, or RCTs with a low risk of bias
55.Chen J, Chen Z, Yan Q, Yin R, Feng Y, Wang L. Patient-reported outcome measures comparing povidone iodine rinse and chlorhexidine rinse for dental implant therapy: a randomized controlled trial. BMC Oral Health. 2025;25(1):1233.	Randomised controlled trial	Checklist 2: Controlled Trials	1.1 The study addresses an appropriate and clearly focused question Yes<br>1.2 The assignment of subjects to treatment groups is randomised Yes<br>1.3 An adequate concealment method is used Yes<br>1.4 The design keeps subjects and investigators 'blind' about treatment allocation Yes<br>1.5 The treatment and control groups are similar at the start of the trial Yes<br>1.6 The only difference between groups is the treatment under investigation Yes<br>1.7 All relevant outcomes are measured in a standard, valid and reliable way Yes<br>1.8 Percentage of individuals in each arm who dropped out before completion 0%<br>1.9 All subjects analysed in the groups to which they were randomly allocated (ITT) Yes<br>1.10 Where the study is carried out at more than one site, results are comparable for all sites N/A	High quality (++) - SIGN 1++ High quality meta-analyses, systematic reviews of RCTs, or RCTs with a very low risk of bias

Full Citation	Study Design	SIGN Tool	Item and response	Overall Risk of Bias / Confidence Rating
56.Mahaseni Aghdam H, Kasirolvalad S, Lasemi E, Farahmand M, Ardalani R, Shalalvand M. Comparative effects of hydrogen peroxide (Nanosil) and chlorhexidine mouthwashes on healing and pain after impacted mandibular third molar surgery: a randomized clinical trial. BMC Oral Health. 2025;25(1):1426.	Randomised clinical trial	Checklist 2: Controlled Trials	1.1 The study addresses an appropriate and clearly focused question Yes<br>1.2 The assignment of subjects to treatment groups is randomised Yes<br>1.3 An adequate concealment method is used Yes<br>1.4 The design keeps subjects and investigators 'blind' about treatment allocation Yes<br>1.5 The treatment and control groups are similar at the start of the trial Yes<br>1.6 The only difference between groups is the treatment under investigation Can't say<br>1.7 All relevant outcomes are measured in a standard, valid and reliable way Can't say<br>1.8 Percentage of individuals in each arm who dropped out before completion 0%<br>1.9 All subjects analysed in the groups to which they were randomly allocated (ITT) Yes<br>1.10 Where the study is carried out at more than one site, results are comparable for all sites N/A	Acceptable (+) - SIGN 1+ Well-conducted meta-analyses, systematic reviews of RCTs, or RCTs with a low risk of bias
**Block 4 · Management of postoperative complications**				
57.Sanz-Sánchez I, Sanz-Martín I, Ortiz-Vigón A, Molina A, Sanz M. Complications in bone-grafting procedures: Classification and management. Periodontol 2000. 2022;88(1):86–102.	Narrative expert review	No SIGN methodology checklist applicable		
58.Barboza EP, Caúla AL. Diagnoses, clinical classification, and proposed treatment of spontaneous early exposure of submerged implants. Implant Dent. 2002;11(4):331–7.	Retrospective case series	No SIGN methodology checklist applicable		
59.Chiapasco M, Zaniboni M. Clinical outcomes of GBR procedures to correct peri-implant dehiscences and fenestrations: a systematic review. Clin Oral Implants Res. 2009;20 Suppl 4:113–23.	Systematic review	Checklist 1: Systematic Reviews and Meta-Analyses	1.1 The research question is clearly defined and inclusion/exclusion criteria are listed Yes<br>1.2 A comprehensive literature search is carried out Yes<br>1.3 At least two people should have selected studies Yes<br>1.4 At least two people should have extracted data Can't say<br>1.5 Grey/unpublished data was sought No<br>1.6 The excluded studies are listed Yes<br>1.7 The relevant characteristics of the included studies are provided Yes	Low quality (−) - SIGN 1− Meta-analyses, systematic reviews of RCTs, or RCTs with a high risk of bias

Full Citation	Study Design	SIGN Tool	Item and response	Overall Risk of Bias / Confidence Rating
			1.8 The scientific quality of the included studies was assessed and reported No<br>1.9 Was the scientific quality of the included studies used appropriately? No<br>1.10 Appropriate methods are used to combine the individual study findings N/A<br>1.11 The likelihood of publication bias was assessed appropriately No<br>1.12 Conflicts of interest are declared Yes	
60.Kim YK, Yun PY. Risk Factors for Wound Dehiscence after Guided Bone Regeneration in Dental Implant Surgery. Maxillofac Plast Reconstr Surg. 2014;36(3):116–23.	Retrospective observational study	Checklist 3: Cohort Studies	1.1 The study addresses an appropriate and clearly focused question Yes<br>1.2 The two groups are selected from source populations comparable in all respects other than the factor under investigation Yes<br>1.3 The study indicates how many of the people asked to take part did so, in each group Yes<br>1.4 Likelihood that eligible subjects might have the outcome at enrolment is assessed and taken into account No<br>1.5 Percentage of individuals recruited into each arm who dropped out before study completion N/A<br>1.6 Comparison is made between full participants and those lost to follow up, by exposure status N/A<br>1.7 The outcomes are clearly defined Yes<br>1.8 The assessment of outcome is made blind to exposure status No<br>1.9 Where blinding was not possible, there is recognition that knowledge of exposure status could have influenced outcome assessment Yes<br>1.10 The method of assessment of exposure is reliable Yes<br>1.11 Evidence from other sources is used to demonstrate validity and reliability of outcome assessment No<br>1.12 Exposure level or prognostic factor is assessed more than once No<br>1.13 The main potential confounders are identified and taken into account in the design and analysis No<br>1.14 Have confidence intervals been provided? Yes	Low quality (−) - SIGN 2− Cohort or case-control studies with a high risk of bias
61.Scheyer ET, McGuire MK. Evaluation of Premature Membrane Exposure and Early Healing in Guided Bone Regeneration of Peri-Implant Dehiscence and Fenestration Defects With a Slowly Resorbing Porcine Collagen Ribose Cross-	Prospective consecutive case series	No SIGN methodology checklist applicable		

Full Citation	Study Design	SIGN Tool	Item and response	Overall Risk of Bias / Confidence Rating
Linked Membrane: A Consecutive Case Series. Clin Adv Periodontics. 2015;5(3):165–70.				
62.Tarakji B, Saleh LA, Umair A, Azzeghaiby SN, Hanouneh S. Systemic review of dry socket: aetiology, treatment, and prevention. J Clin Diagn Res. 2015;9(4):ZE10-13.	Narrative review	No SIGN methodology checklist applicable		
63.Rodríguez Sánchez F, Rodríguez Andrés C, Arteagoitia Calvo I. Does Chlorhexidine Prevent Alveolar Osteitis After Third Molar Extractions? Systematic Review and Meta-Analysis. J Oral Maxillofac Surg. 2017;75(5):901–14.	Systematic review and meta-analysis	Checklist 1: Systematic Reviews and Meta-Analyses	1.1 The research question is clearly defined and inclusion/exclusion criteria are listed Yes<br>1.2 A comprehensive literature search is carried out Yes<br>1.3 At least two people should have selected studies Yes<br>1.4 At least two people should have extracted data Yes<br>1.5 Grey/unpublished data was sought Yes<br>1.6 The excluded studies are listed Yes<br>1.7 The relevant characteristics of the included studies are provided Yes<br>1.8 The scientific quality of the included studies was assessed and reported Yes<br>1.9 Was the scientific quality of the included studies used appropriately? Yes<br>1.10 Appropriate methods are used to combine the individual study findings Yes<br>1.11 The likelihood of publication bias was assessed appropriately Yes<br>1.12 Conflicts of interest are declared Yes	Acceptable (+) - SIGN 1+ Well-conducted meta-analyses, systematic reviews of RCTs, or RCTs with a low risk of bias
64.Taberner-Vallverdú M, Sánchez-Garcés MÁ, Gay-Escoda C. Efficacy of different methods used for dry socket prevention and risk factor analysis: A systematic review. Med Oral Patol Oral Cir Bucal. 2017;22(6):e750–8.	Systematic review	Checklist 1: Systematic Reviews and Meta-Analyses	1.1 The research question is clearly defined and inclusion/exclusion criteria are listed Yes<br>1.2 A comprehensive literature search is carried out Yes<br>1.3 At least two people should have selected studies No<br>1.4 At least two people should have extracted data No<br>1.5 Grey/unpublished data was sought No<br>1.6 The excluded studies are listed No<br>1.7 The relevant characteristics of the included studies are provided Yes	Low quality (−) - SIGN 1− Meta-analyses, systematic reviews of RCTs, or RCTs with a high risk of bias

Full Citation	Study Design	SIGN Tool	Item and response	Overall Risk of Bias / Confidence Rating
			1.8 The scientific quality of the included studies was assessed and reported Yes<br>1.9 Was the scientific quality of the included studies used appropriately? Yes<br>1.10 Appropriate methods are used to combine the individual study findings N/A<br>1.11 The likelihood of publication bias was assessed appropriately No<br>1.12 Conflicts of interest are declared Yes	
65.Chye RML, Perrotti V, Piattelli A, Iaculli F, Quaranta A. Effectiveness of Different Commercial Chlorhexidine-Based Mouthwashes After Periodontal and Implant Surgery: A Systematic Review. Implant Dent. 2019;28(1):74–85.	Systematic review	Checklist 1: Systematic Reviews and Meta-Analyses	1.1 The research question is clearly defined and inclusion/exclusion criteria are listed Yes<br>1.2 A comprehensive literature search is carried out Yes<br>1.3 At least two people should have selected studies Yes<br>1.4 At least two people should have extracted data Yes<br>1.5 Grey/unpublished data was sought No<br>1.6 The excluded studies are listed Yes<br>1.7 The relevant characteristics of the included studies are provided Yes<br>1.8 The scientific quality of the included studies was assessed and reported Yes<br>1.9 Was the scientific quality of the included studies used appropriately? Yes<br>1.10 Appropriate methods are used to combine the individual study findings N/A<br>1.11 The likelihood of publication bias was assessed appropriately No<br>1.12 Conflicts of interest are declared Yes	Acceptable (+) - SIGN 1+ Well-conducted meta-analyses, systematic reviews of RCTs, or RCTs with a low risk of bias
66.Tay JRH, Lu XJ, Lai WMC, Fu JH. Clinical and histological sequelae of surgical complications in horizontal guided bone regeneration: a systematic review and proposal for management. Int J Implant Dent. 2020;6(1):76.	Systematic review and meta-analysis	Checklist 1: Systematic Reviews and Meta-Analyses	1.1 The research question is clearly defined and inclusion/exclusion criteria are listed Yes<br>1.2 A comprehensive literature search is carried out Yes<br>1.3 At least two people should have selected studies Yes<br>1.4 At least two people should have extracted data Yes<br>1.5 Grey/unpublished data was sought No<br>1.6 The excluded studies are listed Yes<br>1.7 The relevant characteristics of the included studies are provided Yes<br>1.8 The scientific quality of the included studies was assessed and reported Yes<br>1.9 Was the scientific quality of the included studies used appropriately? Yes	Low quality (−) - SIGN 1− Meta-analyses, systematic reviews of RCTs, or RCTs with a high risk of bias

Full Citation	Study Design	SIGN Tool	Item and response	Overall Risk of Bias / Confidence Rating
			1.10 Appropriate methods are used to combine the individual study findings Yes<br>1.11 The likelihood of publication bias was assessed appropriately Yes<br>1.12 Conflicts of interest are declared Yes	
67.Cunha G, Carvalho PH de A, Quirino LC, Torres LHS, Filho VAP, Gabrielli MFR, et al. Titanium Mesh Exposure After Bone Grafting: Treatment Approaches-A Systematic Review. Craniomaxillofac Trauma Reconstr. 2022;15(4):397–405.	Systematic review	Checklist 1: Systematic Reviews and Meta-Analyses	1.1 The research question is clearly defined and inclusion/exclusion criteria are listed Yes<br>1.2 A comprehensive literature search is carried out Yes<br>1.3 At least two people should have selected studies Yes<br>1.4 At least two people should have extracted data Yes<br>1.5 Grey/unpublished data was sought No<br>1.6 The excluded studies are listed No<br>1.7 The relevant characteristics of the included studies are provided Yes<br>1.8 The scientific quality of the included studies was assessed and reported Yes<br>1.9 Was the scientific quality of the included studies used appropriately? Can't say<br>1.10 Appropriate methods are used to combine the individual study findings N/A<br>1.11 The likelihood of publication bias was assessed appropriately No<br>1.12 Conflicts of interest are declared Yes	Low quality (−) - SIGN 1− Meta-analyses, systematic reviews of RCTs, or RCTs with a high risk of bias
68.Urban I, Sanz-Sánchez I, Monje A, Montero E. Complications and treatment errors in peri-implant hard tissue management. Periodontol 2000. 2023;92(1):278–98.	Narrative expert review	No SIGN methodology checklist applicable		
69.Nascimento-Júnior EM, Costa FW, Martins-Filho PR. Certainty of evidence on the effects of cryotherapy, surgical wound closure, and chlorhexidine on clinical and patient-centered outcomes after third molar surgery: evidence mapping of systematic reviews and meta-analyses. Med Oral Patol Oral Cir Bucal. 2025;30(2):e199–210.	Evidence mapping of systematic reviews and meta-analyses	Checklist 1: Systematic Reviews and Meta-Analyses	1.1 The research question is clearly defined and inclusion/exclusion criteria are listed Yes<br>1.2 A comprehensive literature search is carried out Yes<br>1.3 At least two people should have selected studies Yes<br>1.4 At least two people should have extracted data Yes<br>1.5 Grey/unpublished data was sought No<br>1.6 The excluded studies are listed Yes<br>1.7 The relevant characteristics of the included studies are provided Yes<br>1.8 The scientific quality of the included studies was assessed and reported Yes	Acceptable (+) - SIGN 1+ Well-conducted meta-analyses, systematic reviews of RCTs, or RCTs with a low risk of bias

Full Citation	Study Design	SIGN Tool	Item and response	Overall Risk of Bias / Confidence Rating
			1.9 Was the scientific quality of the included studies used appropriately? Yes<br>1.10 Appropriate methods are used to combine the individual study findings Yes<br>1.11 The likelihood of publication bias was assessed appropriately N/A<br>1.12 Conflicts of interest are declared Yes	
**Block 5 · Future perspectives and research priorities**				
70.Shahakbari R, Eshghpour M, Rajaei A, Rezaei NM, Golfakhrabadi P, Nejat A. Effectiveness of green tea mouthwash in comparison to chlorhexidine mouthwash in patients with acute pericoronitis: a randomized clinical trial. Int J Oral Maxillofac Surg. 2014;43(11):1394–8.	Randomised clinical trial	Checklist 2: Controlled Trials	1.1 The study addresses an appropriate and clearly focused question Yes<br>1.2 The assignment of subjects to treatment groups is randomised Yes<br>1.3 An adequate concealment method is used Yes<br>1.4 The design keeps subjects and investigators 'blind' about treatment allocation Yes<br>1.5 The treatment and control groups are similar at the start of the trial Yes<br>1.6 The only difference between groups is the treatment under investigation Yes<br>1.7 All relevant outcomes are measured in a standard, valid and reliable way Yes<br>1.8 Percentage of individuals in each arm who dropped out before completion 4%<br>1.9 All subjects analysed in the groups to which they were randomly allocated (ITT) No<br>1.10 Where the study is carried out at more than one site, results are comparable for all sites N/A	Acceptable (+) - SIGN 1+ Well-conducted meta-analyses, systematic reviews of RCTs, or RCTs with a low risk of bias
71.Reitzel RA, Rosenblatt J, Gerges BZ, Jarjour A, Fernández-Cruz A, Raad II. The potential for developing new antimicrobial resistance from the use of medical devices containing chlorhexidine, minocycline, rifampicin and their combinations: a systematic review. JAC Antimicrob Resist. 2020;2(1):dlaa002.	Systematic review	Checklist 1: Systematic Reviews and Meta-Analyses	1.1 The research question is clearly defined and inclusion/exclusion criteria are listed Yes<br>1.2 A comprehensive literature search is carried out Yes<br>1.3 At least two people should have selected studies Yes<br>1.4 At least two people should have extracted data Can't say<br>1.5 Grey/unpublished data was sought No<br>1.6 The excluded studies are listed No<br>1.7 The relevant characteristics of the included studies are provided Yes<br>1.8 The scientific quality of the included studies was assessed and reported Yes<br>1.9 Was the scientific quality of the included studies used appropriately? Yes	Acceptable (+) - SIGN 1+ Well-conducted meta-analyses, systematic reviews of RCTs, or RCTs with a low risk of bias

**Full Citation**	**Study Design**	**SIGN Tool**	**Item and response**	**Overall Risk of Bias / Confidence Rating**
			1.10 Appropriate methods are used to combine the individual study findings N/A<br>1.11 The likelihood of publication bias was assessed appropriately No<br>1.12 Conflicts of interest are declared Yes	
72.Rosa CDDRD, Gomes JM de L, Moraes SLD de, Lemos CAA, da Fonte TP, Limirio JPJ de O, et al. Use of chlorhexidine chip after scaling and root planning on periodontal disease: A systematic review and meta-analysis. Saudi Dent J. 2021;33(1):1–10.	Systematic review and meta-analysis	Checklist 1: Systematic Reviews and Meta-Analyses	1.1 The research question is clearly defined and inclusion/exclusion criteria are listed Yes<br>1.2 A comprehensive literature search is carried out Yes<br>1.3 At least two people should have selected studies Yes<br>1.4 At least two people should have extracted data Yes<br>1.5 Grey/unpublished data was sought No<br>1.6 The excluded studies are listed Yes<br>1.7 The relevant characteristics of the included studies are provided Yes<br>1.8 The scientific quality of the included studies was assessed and reported Yes<br>1.9 Was the scientific quality of the included studies used appropriately? Yes<br>1.10 Appropriate methods are used to combine the individual study findings Yes<br>1.11 The likelihood of publication bias was assessed appropriately No<br>1.12 Conflicts of interest are declared Yes	Acceptable (+) - SIGN 1+ Well-conducted meta-analyses, systematic reviews of RCTs, or RCTs with a low risk of bias
73.Ibraheem WI, Hakami AA, Shafei AA, Jerah SA, Almarghlani A, Makrami AM, et al. Evaluating Soft Tissue Healing after Implant Placement Using Two Different Mouthwashes (Myrrh and Chlorhexidine Gluconate): A Randomized Control Trial. Medicina (Kaunas). 2022 Sep 26;58(10):1351.	Randomised controlled trial	Checklist 2: Controlled Trials	1.1 The study addresses an appropriate and clearly focused question Yes<br>1.2 The assignment of subjects to treatment groups is randomised Yes<br>1.3 An adequate concealment method is used Yes<br>1.4 The design keeps subjects and investigators 'blind' about treatment allocation Yes<br>1.5 The treatment and control groups are similar at the start of the trial Yes<br>1.6 The only difference between groups is the treatment under investigation Yes<br>1.7 All relevant outcomes are measured in a standard, valid and reliable way Yes<br>1.8 Percentage of individuals in each arm who dropped out before completion 12.5%<br>1.9 All subjects analysed in the groups to which they were randomly allocated (ITT) No<br>1.10 Where the study is carried out at more than one site, results are comparable for all sites N/A	Acceptable (+) - SIGN 1+ Well-conducted meta-analyses, systematic reviews of RCTs, or RCTs with a low risk of bias
